# Arsenic Separation from Arsenic-Bearing Materials and Preparation of Arsenic Products: Recent Advances

**DOI:** 10.3390/ma19214518

**Published:** 2026-10-27

**Authors:** Jizhao Cui, Heng Wang, Xuepeng Li, Jianxun Song, Zepeng Lv, Shaolong Li, Qinghua Yan, Wenchang Wang, Juan Wang, Cheng Luo, Huaping Shen, Xinbing Zhou

**Affiliations:** 1 Dalong Jingheng New Materials Co., Ltd., Tongren 554001, China; 1993136309@163.com (J.C.); 15286733706@163.com (X.L.); 18239433265@163.com (Q.Y.); 19024763790@163.com (W.W.); 19385230170@163.com (J.W.); 15381257954@163.com (C.L.); 18385688689@163.com (H.S.); 18379601714@163.com (X.Z.); 2 College of Materials and Chemical Engineering, Tongren University, Tongren 554300, China; 3 Zhongyuan Key Metals Laboratory, School of Materials Science and Engineering, Zhengzhou University, Zhengzhou 450001, China; jianxun.song@zzu.edu.cn (J.S.); lvzepeng@zzu.edu.cn (Z.L.); lishaolong135@163.com (S.L.)

**Keywords:** arsenic-bearing materials, arsenic separation, pyrometallurgy, hydrometallurgy, combined process of pyrometallurgy and hydrometallurgy, arsenic stabilization, high-purity arsenic, arsenic products

## Abstract

Arsenic is used in compound semiconductors, optoelectronic materials, specialty alloys, and fine chemicals owing to its distinctive chemical and electronic properties. However, arsenic is generated as an associated element in nonferrous-metallurgical and resource-recycling processes, including flue dusts, slags, and aqueous wastes, where its toxicity needs to be properly and safely controlled. This review examines arsenic separation from arsenic-bearing materials and arsenic-product preparation, with emphasis on reaction principles, requirements of raw materials, and the typical processing and preparation techniques. Pyrometallurgical approaches include roasting, vacuum roasting/distillation, reduction smelting, and bath smelting; hydrometallurgical approaches include acidic and alkaline leaching, precipitation-based immobilization, ion exchange, adsorption, and solvent extraction. For complex arsenic-bearing materials, multiple metallurgical methods need to be used for processing. Product-preparation methods are summarized for crude and refined As_2_O_3_, metallic arsenic, high-purity arsenic, AsH_3_, GaAs, InAs, and Zn_3_As_2_. Further work is needed to improve separation selectivity, verify the long-term stability of immobilized arsenic, and refine processes for high-purity arsenic production. Recovery should be limited to applications with defined product specifications and justified demand, rather than expanded into low-value uses.

## 1. Introduction

**Figure 1:**
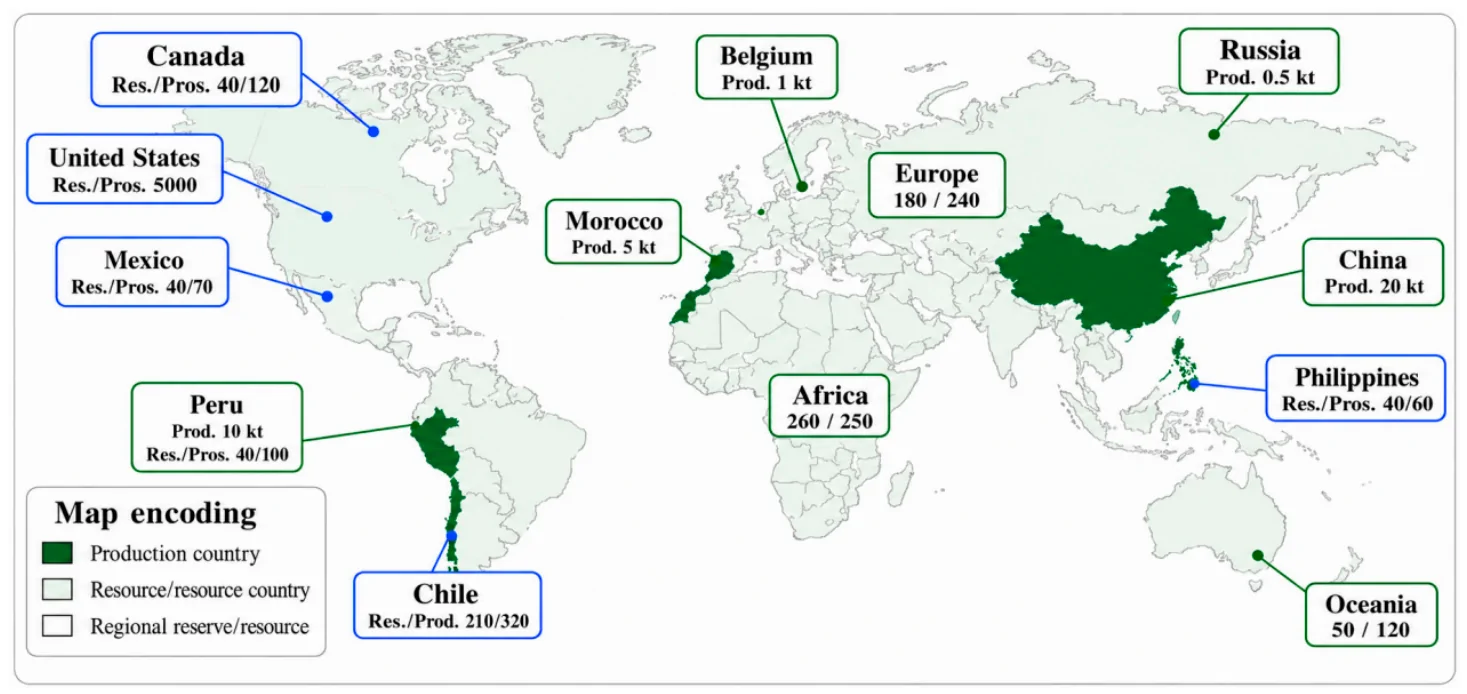
Global distribution of representative natural arsenic occurrences. AI was used for this figure, specifically GPT 5.5, mainly for logical sorting.

Arsenic (As) is a metalloid that occurs as sulfides, oxides, arsenates, or associated constituents in polymetallic minerals and metallurgical by-product streams derived from copper, lead, zinc, gold, cobalt, and other ores. Its chemical reactivity and multiple oxidation states confer value in materials science, chemical synthesis, medicine, and semiconductor technologies. However, traditional high-risk uses, including wood preservatives, pesticides, pigments, and conventional alloy additives, are increasingly restricted [1,2]. High-purity arsenic and its compounds are increasingly used as precursors for compound semiconductors such as GaAs, InAs, and InGaAs. These materials are used in photovoltaic devices, infrared detectors, optoelectronics, wireless communications, aerospace systems, and defense electronics. Arsenic trioxide is also used clinically to treat specific diseases. Arsenic therefore has recoverable value, but any use must account for its toxicity and potential environmental hazard. Representative natural arsenic occurrences are shown in Figure 1.

Arsenic exposure poses substantial environmental and health hazards [3]. In natural and industrial environments, arsenic can migrate and accumulate through groundwater, soils, mining operations, metallurgical flue dusts, waste acids, tailings, and industrial effluents. Inorganic arsenic, particularly As(III), is generally more toxic, mobile, and bioavailable. Long-term exposure through arsenic-bearing water or food may contribute to skin lesions, neurological damage, cardiovascular disease, diabetes, and increased risks of several cancers [4,5]. Because arsenic is persistent, mobile, and subject to complex speciation changes, direct release of untreated arsenic-bearing materials can lead to long-term soil, water, and ecological risks [6]. Safe treatment therefore requires separation, stabilization, controlled disposal, and, where possible, resource recycling or harmless treatment.

Arsenic is generally not mined as a stand-alone primary product. Instead, it becomes enriched as an associated constituent during the mining and smelting of copper, iron, and other ores. Arsenic-bearing copper concentrates, copper-smelting flue dusts, lead–zinc metallurgical flue dusts, arsenic-bearing waste acids, arsenic–alkali slags, arsenic-bearing leachates, and arsenic-bearing tailings are representative by-products or secondary arsenic-bearing resources [7]. Processing larger quantities of complex arsenic-bearing ores increases arsenic recirculation within smelters and produces more arsenic-rich materials [8,9].

**Figure 2:**
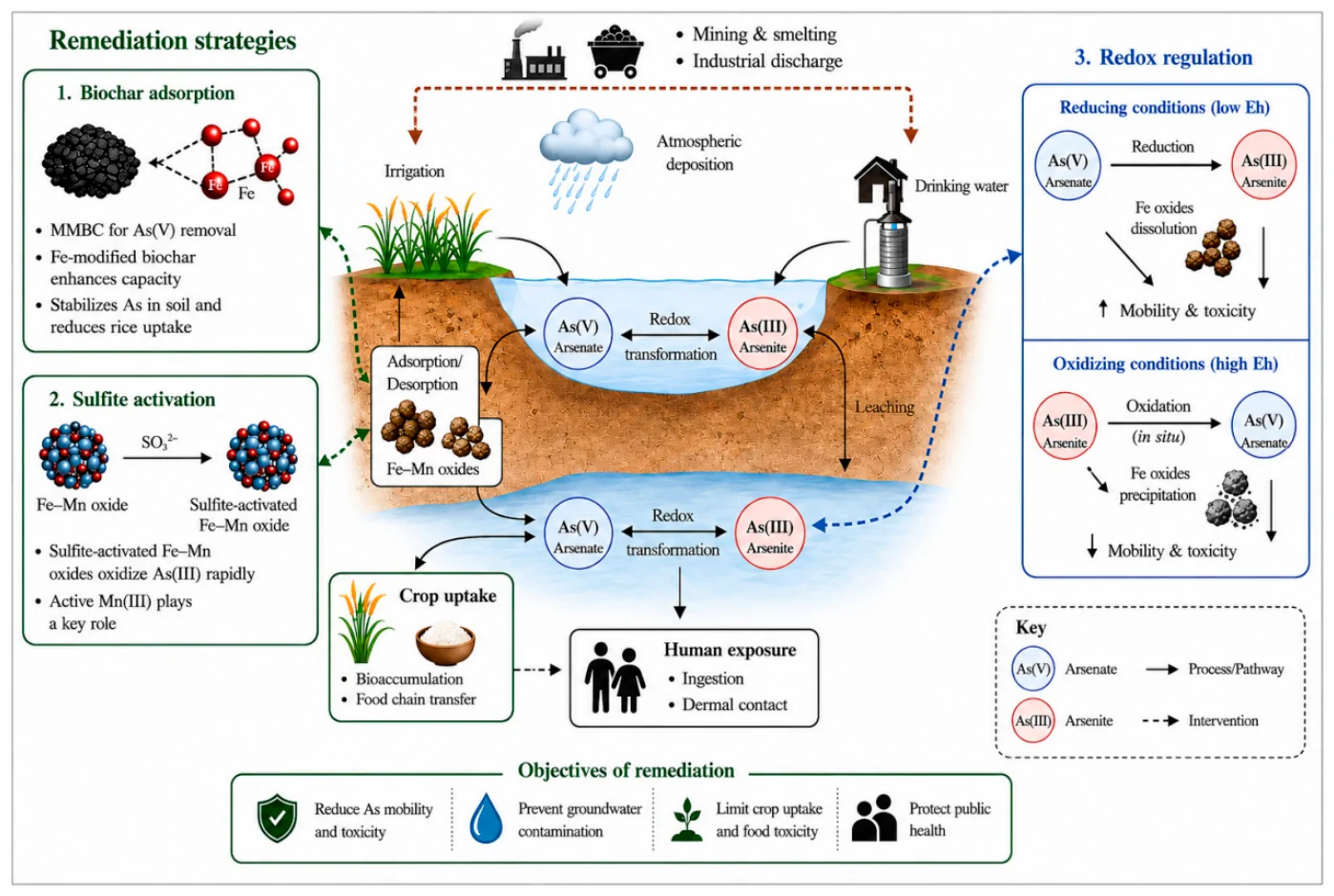
Overview of arsenic separation, stabilization, and product preparation from arsenic-bearing materials. AI was used for this figure, specifically GPT 5.5, mainly for logical sorting.

Recent studies increasingly treat arsenic-bearing materials by sequential separation and enrichment, stabilization, and product conversion rather than by single-step removal [10]. The corresponding process is illustrated in Figure 2. Pyrometallurgical routes are suited to high-temperature volatilization, enrichment, and slag-phase control. Hydrometallurgical routes place greater emphasis on controlling arsenic oxidation state, complexation, and selectivity relative to coexisting metals. Adsorption, membrane separation, and electrochemical methods are generally used for low-concentration polishing [11,12]. These options must be compared in relation to material type, arsenic separation and enrichment, stabilization, and product preparation.

The arsenic products considered in this review include arsenic trioxide, metallic arsenic, high-purity arsenic, arsine, and arsenide semiconductors. Their potential uses include specific medical and chemical applications, optoelectronic devices, infrared detection, and compound semiconductor materials [13,14]. Because of arsenic toxicity, product development is best restricted to high-purity or functional materials produced and used under controlled conditions.

This review examines arsenic separation technologies, stabilization, and the preparation and purification of arsenic products from arsenic-bearing materials. The comparison covers pyrometallurgical, hydrometallurgical, and combined processes and relates arsenic separation to the preparation of As_2_O_3_, metallic arsenic, high-purity arsenic, arsine, and selected arsenides. It also examines the technical, environmental, market, and product-quality limits on arsenic recovery. For each process, the review introduced reactions, material requirements, operating variables, advantages, and limitations [15].

Recent studies combine arsenic separation with recovery or long-term stabilization [16]. Adsorption, precipitation, oxidation, and immobilization have advanced, but their application to metallurgical feeds is still limited by poor selectivity, residue stability, secondary waste generation, and scale-up costs [17]. Current work concentrates on materials and process combinations that separate arsenic from complex matrices, produce stable residues, and recover defined arsenic products where purification is practicable.

The objectives of arsenic treatment should be clearly distinguished as separation, enrichment, recovery, stabilization, and final disposal. Arsenic separation refers to the transfer of arsenic from complex materials into another phase, such as dust or solution, while enrichment indicates an increase in arsenic concentration within a specific process. These processes do not necessarily represent arsenic recovery. Arsenic recovery requires further purification and conversion into products with defined quality requirements. In contrast, stabilization aims to reduce arsenic mobility and environmental risk through the formation of stable phases, whereas final disposal involves the controlled management of unrecoverable arsenic-containing residues.

## 2. Natural Occurrence and Mineral Sources of Arsenic

Arsenic occurs naturally in a variety of sulfide, sulfosalt, and arsenide minerals. Arsenopyrite (FeAsS) is one of the most abundant arsenic-bearing minerals, while realgar (As_4_S_4_) and orpiment (As_2_S_3_) represent important arsenic-rich mineral sources. Arsenic is also commonly associated with polymetallic ores in minerals such as enargite (Cu_3_AsS_4_) and tennantite, particularly in Cu–Au and other sulfide deposits. Arsenic is consequently recovered both from primary arsenic minerals and as a by-product of mining and processing arsenic-bearing nonferrous-metal ores.

The mineralogical occurrence and oxidation state of arsenic strongly influence its thermal reactivity, aqueous speciation, and subsequent separation or stabilization behavior. In formal valence terms, arsenic in arsenopyrite (FeAsS), realgar (As_4_S_4_), and orpiment (As_2_S_3_) is commonly represented as As(I), As(II), and As(III), respectively, whereas arsenic in enargite (Cu_3_AsS_4_) is commonly described using a higher formal oxidation state, although the electronic structure of complex sulfide minerals may deviate from a simple ionic-valence description. These differences mean that total arsenic content alone is insufficient for process selection; mineral phase, arsenic valence, coexisting metals, and the intended final destination of arsenic must also be considered [18].

Oxidative roasting converts sulfidic arsenic phases such as arsenopyrite, realgar, and orpiment into volatile arsenic oxides, principally As_2_O_3_/As_4_O_6_; collection of the condensed dust separates and enriches arsenic. In hydrometallurgical systems, oxidative leaching can instead transfer arsenic into the solution as As(III) or As(V). Cu–As sulfosalts such as enargite require simultaneous control of copper recovery and arsenic partitioning. Arsenic oxides are generally more amenable to aqueous dissolution and subsequent purification, whereas arsenate phases may require acidic or alkaline conversion, reduction, or direct stabilization depending on whether arsenic recovery or final immobilization is desired. Arsenic-bearing alloys are better suited to vacuum-distillation or phase-transformation routes because separation can exploit differences in vapor pressure and phase partitioning. The relationship between arsenic occurrence, speciation, process selection, and final product is summarized in Table 1.

## 3. Arsenic Separation Technologies for Arsenic-Bearing Materials

### 3.1. Pyrometallurgical Separation Technologies

Pyrometallurgical separation exploits the propensity of arsenic, its oxides, sulfides, and elemental arsenic to volatilize, sublime, or partition among phases at elevated temperature. Arsenic can therefore be transferred selectively among the bulk-metal, slag, and off-gas phases. Compared with hydrometallurgical routes, pyrometallurgical processes offer high throughput and are suitable for arsenic-rich solid feedstocks and metallurgical intermediates; however, their effectiveness depends strongly on oxygen potential, the temperature field, off-gas condensation, and secondary pollution control [21]. Both oxidative and reductive volatilization depend on gas-phase transfer of volatile arsenic oxides or elemental arsenic. Oxidative volatilization emphasizes oxygen-potential control, whereas reductive volatilization is more suitable for converting high-valent arsenates or arsenic oxides under reducing conditions [22,23]. Vacuum roasting/distillation uses vapor-pressure differences and fractional condensation for selective enrichment. By contrast, slag-phase immobilization prioritizes environmental stability but can reduce the scope for subsequent product preparation. Feed mineralogy should be characterized before selecting a pyrometallurgical process because sulfide-, oxide-, arsenate-, and alloy-bound arsenic exhibit different volatilization, oxidation, and phase-partitioning behaviors.

#### 3.1.1. Multiple-Hearth Roasting for Arsenic Removal

**Figure 3:**
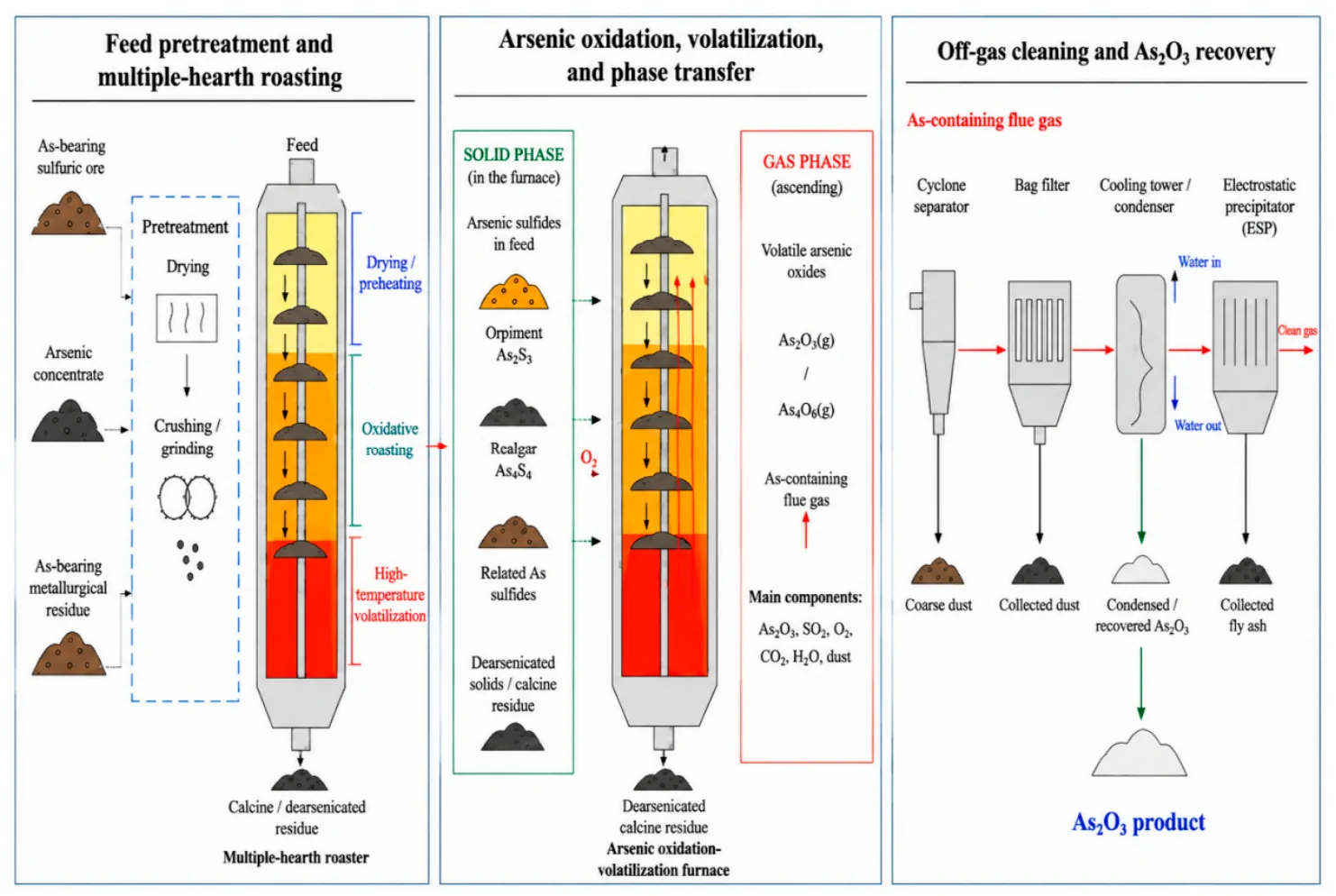
Schematic process flow for multiple-hearth roasting arsenic removal. AI was used for this figure, specifically GPT 5.5, mainly for logical sorting.

Multiple-hearth roasting is a conventional pyrometallurgical route for treating arsenic-bearing sulfide materials, including orpiment, realgar, arsenopyrite, and arsenic-bearing sulfide concentrates. It uses the temperature gradient and atmosphere control across multiple hearths so that solids experience sequential roasting reactions as they descend through the furnace. Under oxidizing conditions, arsenic sulfides such as orpiment (As_2_S_3_) and realgar (As_4_S_4_) are converted to volatile arsenic trioxide (As_2_O_3_), which enters the dust-collection system. The chemical reactions involved in multiple-hearth arsenic removal are listed in Table 2, and a process schematic is shown in Figure 3 [24,25].

As shown in Table 2, oxidation of orpiment and realgar to As_2_O_3_ and SO_2_ is the principal arsenic volatilization pathway in multiple-hearth roasting. The solid As_2_O_3_ formed subsequently sublimes at high temperature to gaseous As_4_O_6_ and migrates with the off-gas to the dust-collection system. During cooling, gaseous As_4_O_6_ condenses to solid As_2_O_3_ and becomes enriched in collected dust. Under excessively oxidizing conditions, part of the As_2_O_3_ may be further oxidized to As_2_O_5_, which can lower arsenic volatilization efficiency [26,27].

In multiple-hearth roasting, the feed is pretreated and passed through staged roasting; the arsenic-bearing off-gas is then cleaned and As_2_O_3_-rich dust is collected [28]. Arsenic-bearing sulfide ores, arsenic concentrates, or metallurgical residues are dried and crushed before entering the multiple-hearth furnace. As the solids descend through the hearths, they undergo preheating, oxidative roasting, and high-temperature volatilization. Orpiment, realgar, and related phases are converted under oxidizing conditions to volatile As_2_O_3_, which enters the off-gas as As_2_O_3(g)_ or As_4_O_6(g),_ while the arsenic-depleted solids form calcine or arsenic-depleted residue [29,30]. The arsenic-bearing off-gas is subsequently treated by cyclone separation, bag filtration, cooling/condensation, and electrostatic precipitation. These operations condense and enrich gas-phase arsenic oxides as As_2_O_3_ while cleaning the off-gas.

#### 3.1.2. Rotary-Kiln Roasting for Arsenic Removal

**Figure 4:**
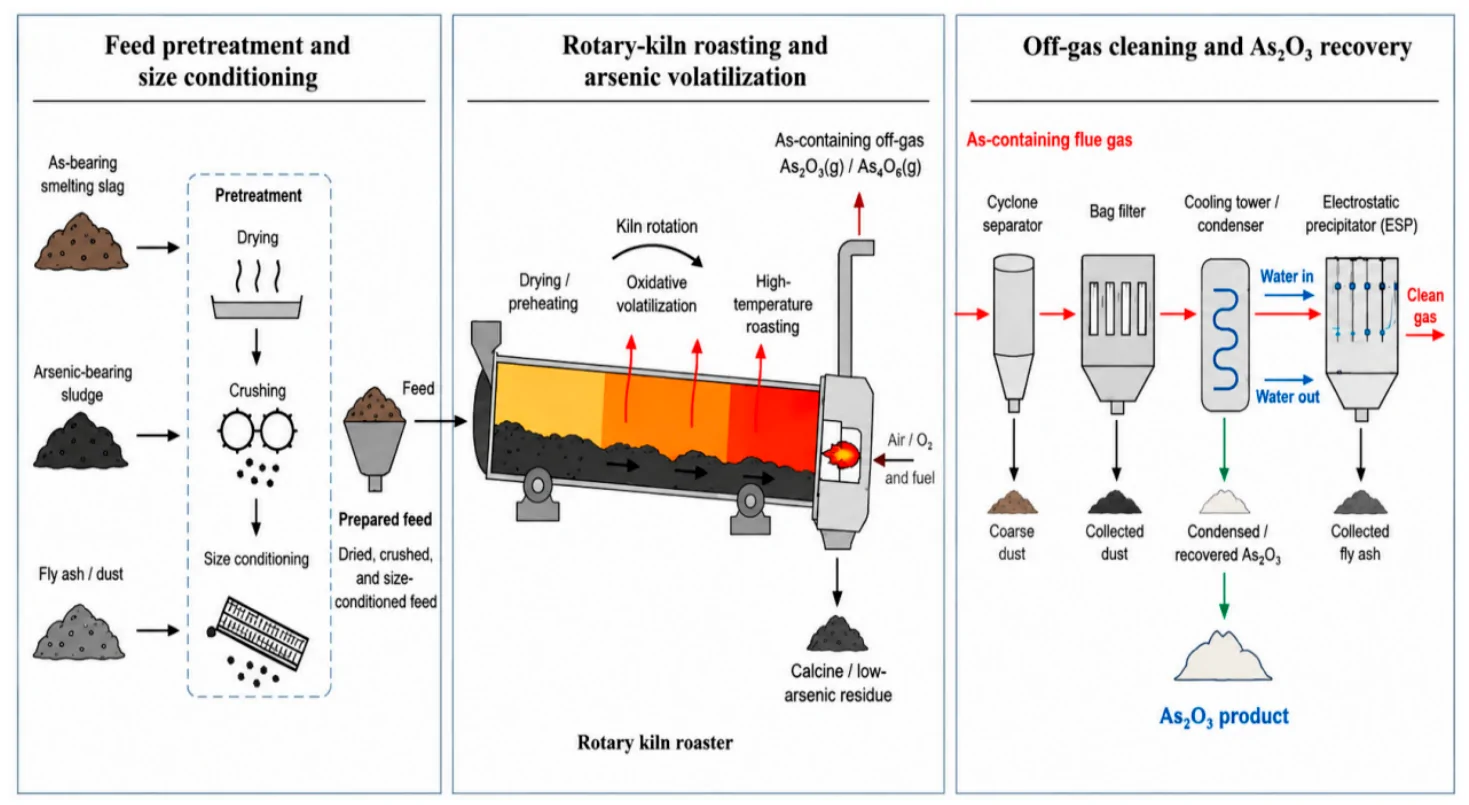
Schematic process flow for rotary-kiln roasting arsenic removal. AI was used for this figure, specifically GPT 5.5, mainly for logical sorting.

Rotary-kiln roasting is a continuous pyrometallurgical arsenic removal technology for arsenic-bearing sulfide ores, metallurgical flue dusts, and arsenic-bearing solid residues. Tumbling, axial solids transport, and zoned temperature control in the kiln allow the feed to undergo successive preheating, oxidation, and volatilization. In an oxidizing atmosphere, arsenic is converted primarily to volatile As_2_O_3_ or As_4_O_6_, which enters the off-gas and is enriched by subsequent condensation and dust collection. Iron, copper, and related components are largely retained in the calcine or kiln residue; this phase partitioning separates arsenic from the retained metals [31,32]. The major reactions are listed in Table 3, and the process flow is illustrated in Figure 4.

As summarized in Table 3, rotary-kiln arsenic removal relies principally on oxidative volatilization of arsenic-bearing sulfides. Orpiment, realgar, arsenopyrite, and related arsenic-bearing phases are converted to As_2_O_3_ in an oxidizing atmosphere; volatile species, including As_4_O_6(g)_, then enter the off-gas and recondense as solid As_2_O_3_ during cooling and particulate collection. Under excessively oxidizing conditions, As_2_O_3_ can be further oxidized to As_2_O_5_ and can react with Fe_2_O_3_ or CaO to form arsenates retained in calcine or kiln residue, thereby reducing arsenic volatilization efficiency [33].

Rotary-kiln treatment consists of feed pretreatment, oxidative volatilization, and recovery of arsenic from the off-gas [34]. Arsenic-bearing smelting slags, sludges, and fly ashes are dried, crushed, and size-conditioned before entering the rotary kiln to improve feed uniformity and roasting performance. As the kiln rotates and maintains axial temperature zones, the feed undergoes drying/preheating, oxidative volatilization, and high-temperature roasting. Arsenic species are converted to volatile As_2_O_3_ or As_4_O_6_ and migrate with the off-gas, while arsenic-depleted solids form calcine or low-arsenic residue [35,36]. Cyclone separation, bag filtration, cooling/condensation, and electrostatic precipitation then condense and enrich arsenic oxides as As_2_O_3_ while cleaning the off-gas.

#### 3.1.3. Vacuum Roasting/Distillation for Arsenic Removal

**Figure 5:**
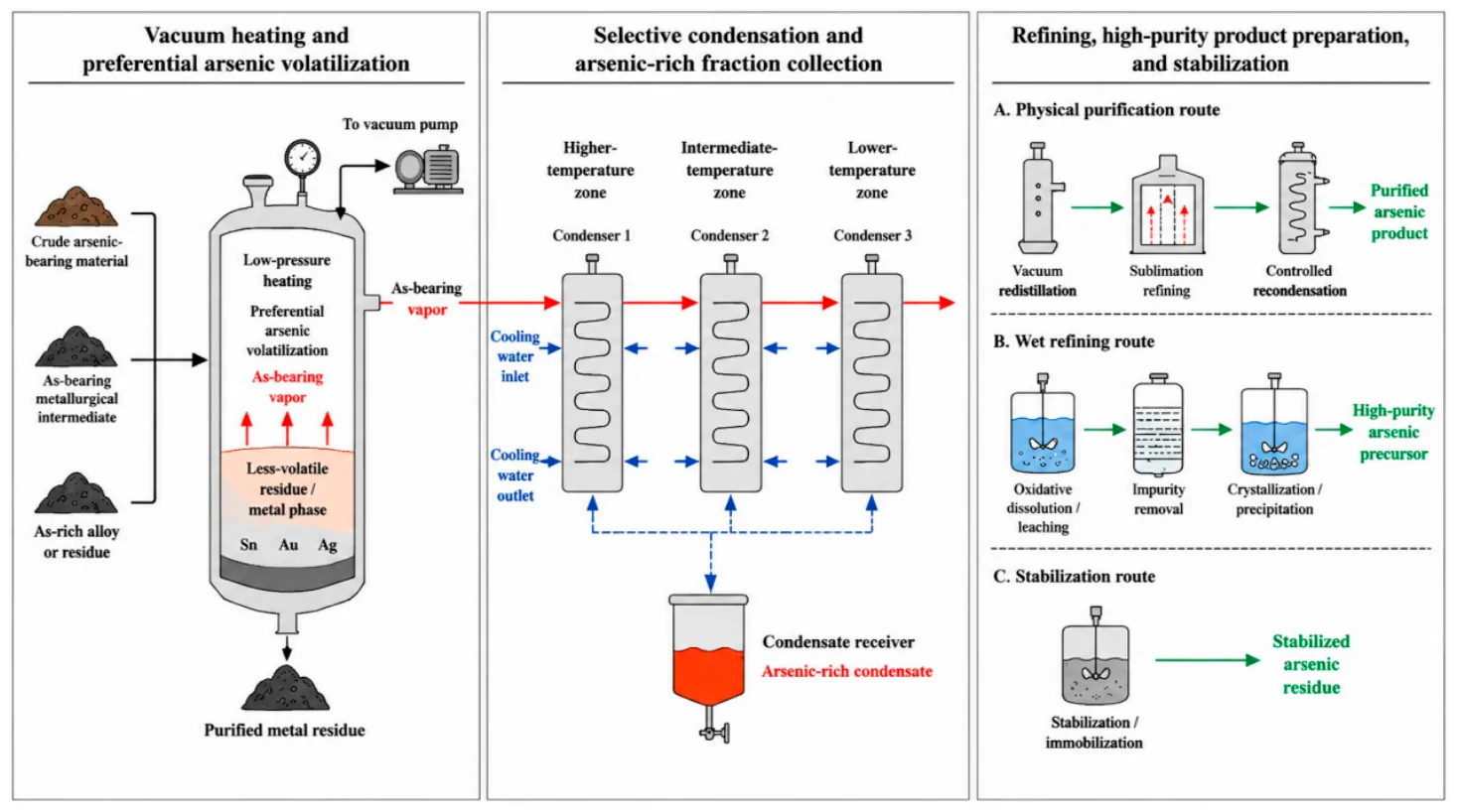
Schematic process flow for vacuum roasting/distillation arsenic removal. AI was used for this figure, specifically GPT 5.5, mainly for logical sorting.

Vacuum roasting/distillation is a pyrometallurgical route that intensifies arsenic volatilization and separation under reduced pressure. It is principally used for arsenic-bearing metallurgical flue dusts, alloys, slags, and related materials. At low pressure or under vacuum, differences in vapor pressure, boiling point, and sublimation temperature between arsenic species and other metal-bearing constituents allow arsenic to volatilize preferentially, whereas relatively nonvolatile copper, iron, nickel, and related components remain in the residue or metal phase. Volatile components such as As_2_O_3_, As_4_O_6_, and elemental arsenic sublime or evaporate at lower temperatures and become enriched in the condensation zone, whereas less volatile coexisting components remain in the residue [37,38]. Major reactions are listed in Table 4, and a process schematic is shown in Figure 5.

As shown in Table 4, vacuum roasting/distillation primarily relies on the preferential volatilization and condensation of arsenic and its compounds at reduced pressure. As_2_O_3_ can sublime to gaseous As_4_O_6_ at elevated temperature and re-form solid As_2_O_3_ in a lower-temperature condenser. Elemental arsenic can form As_4(g)_ and undergo vapor-phase separation. For high-valent arsenic or arsenate-bearing materials, As_2_O_5_ may thermally decompose to As_2_O_3_ and O_2_. Ferric arsenate can first decompose to Fe_2_O_3_ and As_2_O_5_; subsequent thermal decomposition or reduction transfers arsenic to the gas phase [39].

Vacuum roasting/distillation combines low-pressure heating, preferential arsenic volatilization, and fractional condensation. During low-pressure heating, arsenic and its compounds volatilize preferentially because of their comparatively high vapor pressure and low sublimation temperature, whereas relatively nonvolatile tin, gold, silver, and related components remain in the residue or metal phase. The arsenic-bearing vapor enters a condensation and collection train, where temperature gradients and cooling media promote selective condensation and enrichment [40,41].

#### 3.1.4. Smelting Method for Arsenic Removal

**Figure 6:**
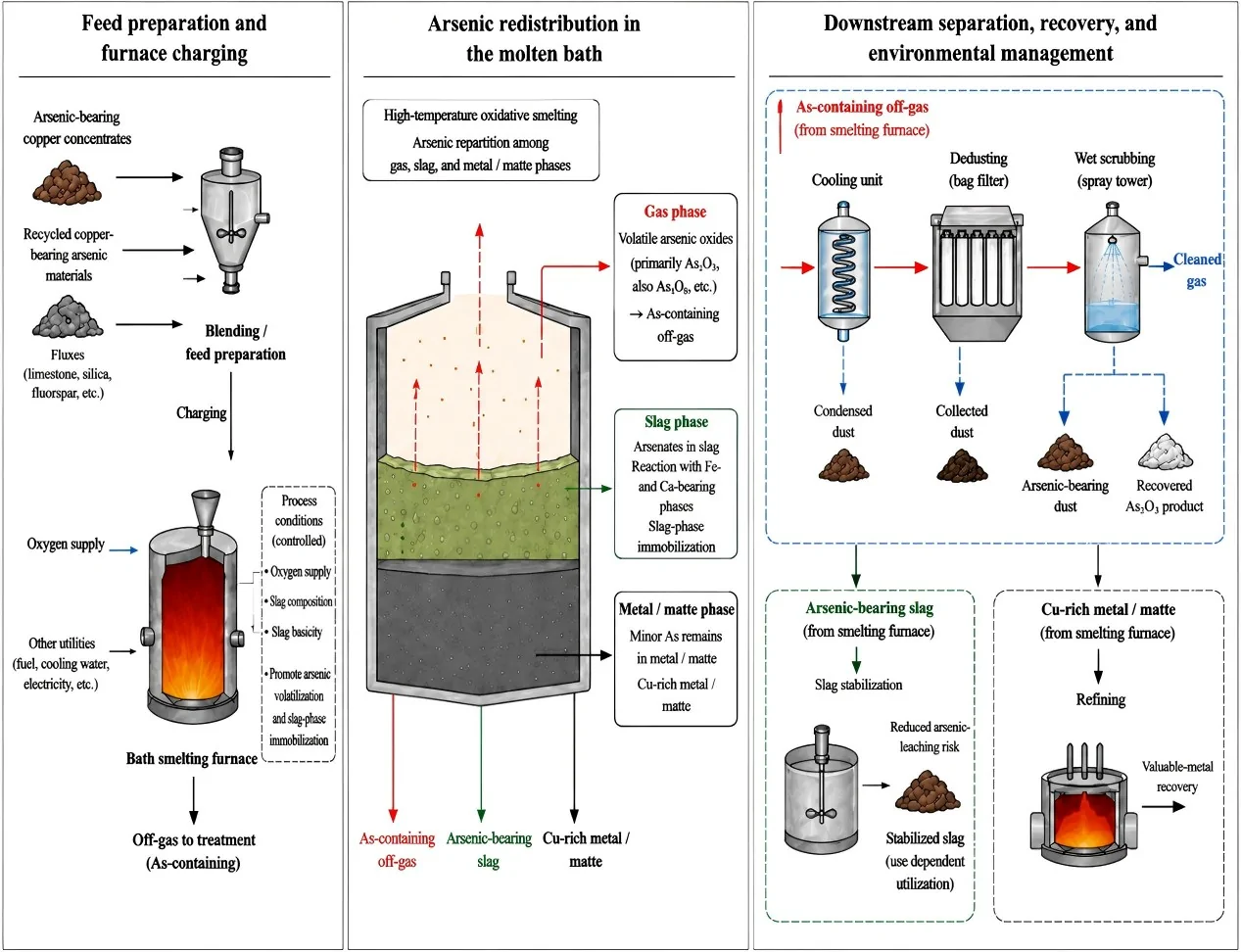
Schematic process flow for bath-smelting arsenic removal. AI was used for this figure, specifically GPT 5.5, mainly for logical sorting.

Smelting method is a high-temperature pyrometallurgical process for arsenic-bearing copper concentrates, metallurgical flue dusts, intermediates, and complex polymetallic feedstocks. Intensive mixing in the molten bath, oxygen-potential control, and gas–slag–metal multiphase partitioning allow arsenic to undergo oxidation, volatilization, or transfer to slag during smelting. Under suitable oxidizing conditions and temperatures, arsenic can be oxidized to volatile As_2_O_3_ or enter the off-gas as species such as As_4_O_6_, where it is enriched by downstream dust collection. A fraction of the arsenic can also react with Fe_2_O_3_, CaO, and related components to form arsenates that enter the slag, thereby separating arsenic from valuable metals such as copper, lead, and zinc [42,43]. Major reactions are listed in Table 5, and the process flow is shown in Figure 6.

As summarized in Table 5, arsenic-bearing minerals during bath smelting can generate volatile As_2_O_3_ under oxidizing conditions. The volatile arsenic species enter the off-gas and become enriched as a solid product during downstream cooling and dust collection. Under stronger oxidation, part of the As_2_O_3_ may be converted to As_2_O_5_ and react with Fe_2_O_3_ or CaO to form relatively stable arsenates in the slag. Slag-forming reactions can therefore influence arsenic partitioning between the gas and slag phases and affect arsenic removal efficiency [44].

The smelting method proceeds through feed preparation, redistribution of arsenic among melt phases, and downstream recovery from gas, slag, or metal processes [45]. For example, arsenic-bearing copper concentrates, recycled copper-bearing arsenic materials, and fluxes are blended and charged to the smelting furnace [44]. Oxygen supply, slag composition, and slag basicity are controlled to promote oxidative volatilization of arsenic and slag-phase immobilization [46]. During high-temperature oxidative smelting, arsenic repartitions among gas, slag, and metal/matte phases: some arsenic enters the off-gas as volatile species such as As_2_O_3_; some reacts with Fe- and Ca-bearing phases to form arsenates in the slag; and a smaller fraction can remain in metal or matte [47,48]. Arsenic-bearing off-gas is cooled, dedusted, and wet-scrubbed to yield arsenic-bearing dust or As_2_O_3_ product. The slag is stabilized according to its arsenic leachability, whereas Cu-rich metal or matte proceeds to refining.

#### 3.1.5. Comparison of Pyrometallurgical Separation Technologies

The quantitative comparison in Table 6 highlights a clear trade-off between process maturity, arsenic removal efficiency, and separation selectivity. The historical multiple-hearth process achieved approximately 94.5% As extraction at 500–700 °C and represents one of the few commercially operated arsenic removal cases [49]. Vacuum distillation provides substantially higher separation performance, with representative As-removal efficiencies reaching 99.43–99.59% at 1353 K and 2 Pa; however, the available evidence remains predominantly laboratory-scale and requires high-cost vacuum equipment [37]. In contrast, the industrial rotary-kiln case demonstrated recovery of >70% Pb and Zn but retained 47.21–72.48% of As in the water-quenched slag, indicating that industrial operation does not necessarily correspond to high arsenic volatilization efficiency [36]. The smelting process can integrate arsenic control with copper recovery, but arsenic distribution among slag, dust, and matte remains strongly dependent on oxygen potential and properties of slag [45]. Process selection therefore depends on feed mineralogy, valuable-metal recovery, arsenic destination, process scale, and secondary waste generation, not on arsenic removal efficiency alone.

### 3.2. Hydrometallurgical Separation Technologies

Because As(III) and As(V) differ in aqueous speciation, adsorption, and precipitation behavior, As(III) is often oxidized to As(V) before ferric precipitation or scorodite crystallization. Hydrometallurgical separation uses the dissolution, precipitation, adsorption, or extraction behavior of arsenic under different acid–base, redox, and complexing conditions to separate arsenic from valuable metals and impurities. Its core operations are leaching, precipitation, and extraction. Acidic, alkaline, neutral, or salt-containing media transfer arsenic from the solid phase to solution. By adjusting pH or adding lime, sulfide reagents, and ferric salts, arsenic can be converted into arsenates, arsenic sulfides, Fe–As precipitates, or scorodite and immobilized in low-solubility solids. Solvent extraction relies on selective complexation and interphase transfer to enrich, purify, or recover specific arsenic species. Relative to pyrometallurgical separation, hydrometallurgical processing operates under milder conditions and provides greater process control. It is suitable for arsenic-bearing waste acids, leachates, flue-dust leachates, and low-concentration arsenic-bearing wastewaters, but reagent consumption, Fe–As sludge stability, and secondary emissions or effluents often determine practical suitability [50,51].

#### 3.2.1. Acidic Leaching

**Figure 7:**
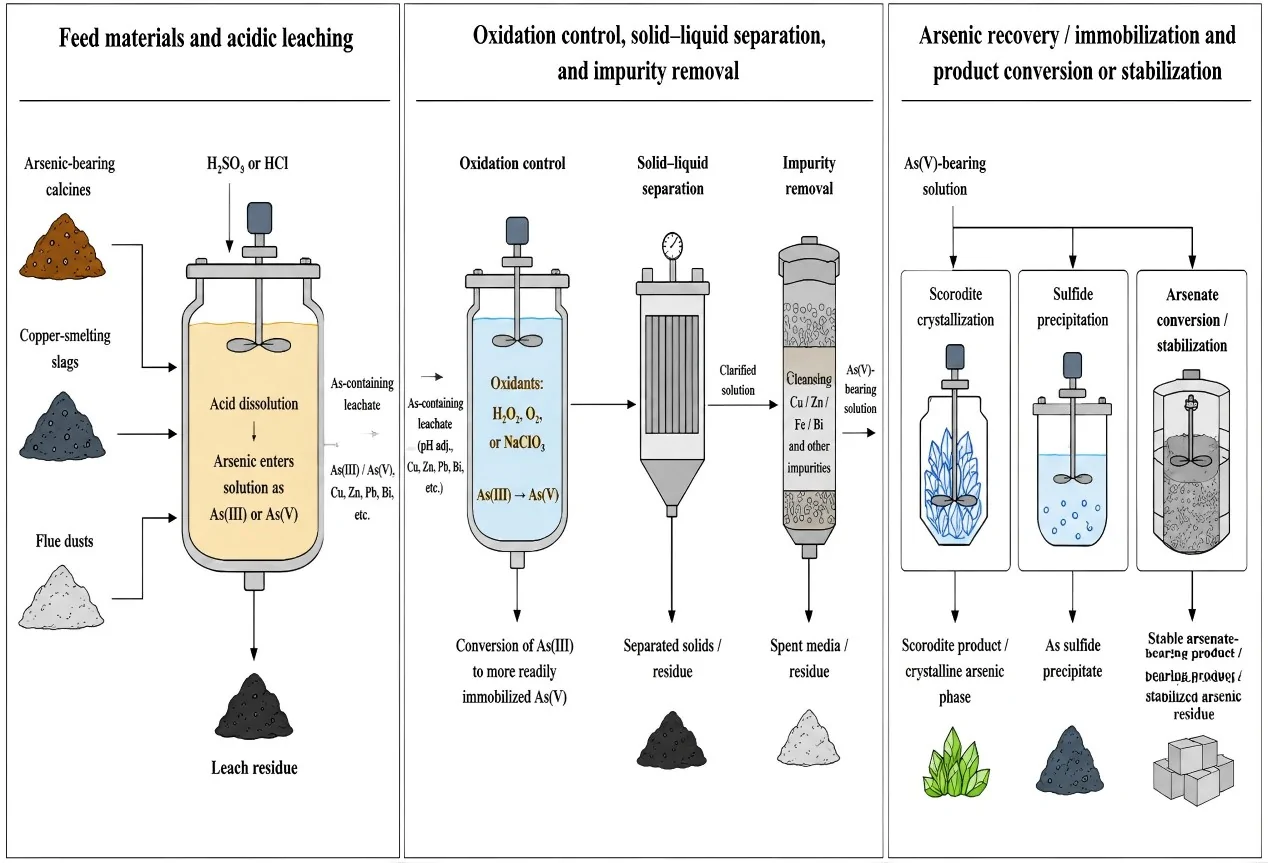
Schematic process flow for acidic leaching and arsenic separation. AI was used for this figure, specifically GPT 5.5, mainly for logical sorting.

Acidic leaching is a common hydrometallurgical route for arsenic-bearing metallurgical flue dusts, sludges, slags, and low-grade arsenic-bearing solid wastes [52,53]. Sulfuric acid, hydrochloric acid, and related acidic media dissolve arsenic-bearing and associated metal-bearing phases, transferring arsenic to the leachate as As(III) or As(V) while retaining part of the sparingly soluble impurity and gangue fraction in the leach residue. Under acidic or oxidative acidic conditions, As_2_O_3_, As_2_O_5_, metal arsenates, and some arsenic sulfides can dissolve or undergo oxidative leaching, thereby transferring arsenic from solid to liquid [54,55]. The principal reactions are listed in Table 7, and a process schematic is provided in Figure 7 [56].

As shown in Table 7, arsenic oxides can undergo hydration or acid dissolution to form H_3_AsO_3_ or H_3_AsO_4_ in solution. Metal arsenates dissolve in the presence of H^+^, releasing metal ions and arsenate species. Arsenic sulfides require oxidants such as O_2_ or Fe^3+^ to undergo oxidative leaching and transfer arsenic from the solid phase to solution. As(III) can subsequently be oxidized to the more readily precipitated and immobilized As(V) form [57].

Acidic leaching dissolves the arsenic-bearing materials, adjusts the arsenic oxidation state, removes insoluble impurities, and directs arsenic to recovery or stabilization [58]. Arsenic-bearing calcines, copper-smelting slags, and flue dusts can be leached with H_2_SO_4_ or HCl so that arsenic enters the solution as As(III) or As(V). Oxidants such as H_2_O_2_, O_2_, or NaClO_3_ can subsequently convert As(III) to As(V), which is more readily precipitated or immobilized [59]. Compared with simple acid dissolution, oxidative acidic leaching is better suited to subsequent scorodite crystallization, sulfide precipitation, or arsenate conversion [60]; its performance nevertheless depends on the acidity, oxidant addition, coexistence of Cu/Zn/Pb/Bi and other impurities, and solid–liquid separation efficiency [61].

#### 3.2.2. Alkaline Leaching

**Figure 8:**
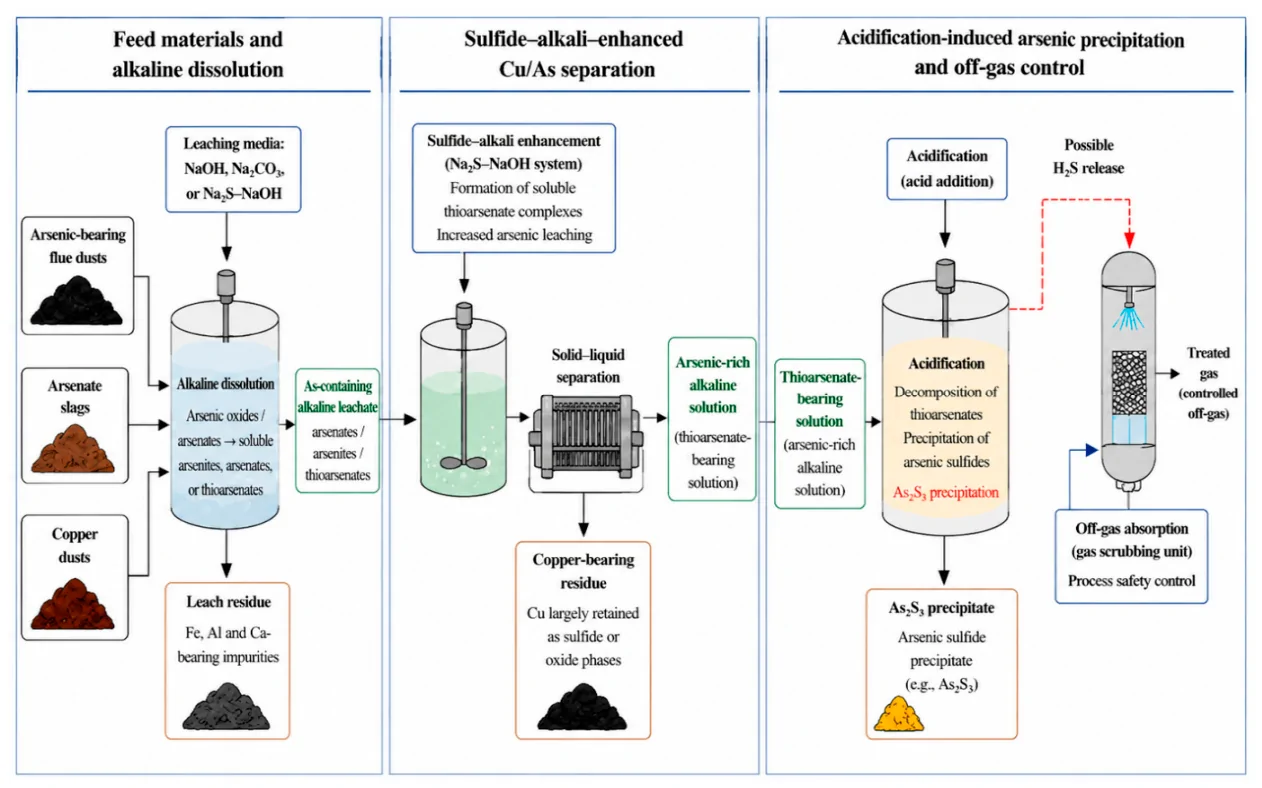
Schematic process flow for alkaline leaching and arsenic separation. AI was used for this figure, specifically GPT 5.5, mainly for logical sorting.

Alkaline leaching is commonly used for arsenic-bearing flue dusts, sludges, arsenate slags, and some arsenic-bearing solid wastes that are difficult to dissolve in acid. Alkaline media such as NaOH, Na_2_CO_3_, and Na_2_S promote dissolution, complexation, or transformation of arsenic-bearing phases, causing arsenic to enter solution as arsenite or arsenate while relatively insoluble Fe-, Ca-, and Si-bearing components remain in the leach residue. Under alkaline conditions, As_2_O_3_, As_2_O_5_, and some metal arsenates can be converted to soluble arsenites or arsenates and thus transfer arsenic selectively from the solid phase to solution [62,63]. Major reactions are listed in Table 8, and the process flow is shown in Figure 8 [64].

As shown in Table 8, As_2_O_3_ and As_2_O_5_ react with NaOH to form soluble sodium arsenite and sodium arsenate, respectively. H_3_AsO_3_ and H_3_AsO_4_ in solution can also be neutralized to the corresponding sodium salts. Metal arsenates, including FeAsO_4_ and Ca_3_(AsO_4_)_2_, can undergo alkaline leaching or conversion in NaOH or Na_2_CO_3_ systems, transferring arsenic to the liquid phase as Na_3_AsO_4_ while Fe and Ca are retained as Fe(OH)_3_ and CaCO_3_ precipitates. For arsenic sulfides, NaOH or Na_2_S systems can form soluble thioarsenate or arsenite species; under oxidizing conditions, these species may be further converted to arsenates [65,66].

Alkaline dissolution may be followed by sulfide–alkali Cu/As separation and acidification to precipitate arsenic. Arsenic-bearing flue dusts, arsenate slags, and copper-smelting flue dusts are leached in NaOH, Na_2_CO_3_, or Na_2_S–NaOH media, converting arsenic oxides or arsenates into soluble arsenites, arsenates, or thioarsenates while many Fe, Al, and Ca-bearing impurities remain in the leach residue [67]. In sulfide–alkali systems, arsenic can further form soluble thioarsenate complexes, increasing arsenic leaching, whereas copper is largely retained as sulfide or oxide phases and is thus separated from arsenic [68]. Subsequent acidification decomposes thioarsenates and precipitates arsenic sulfides such as As_2_S_3_. Because H_2_S may be released, off-gas absorption and process-safety controls are required.

#### 3.2.3. Hot-Water/Neutral Leaching

**Figure 9:**
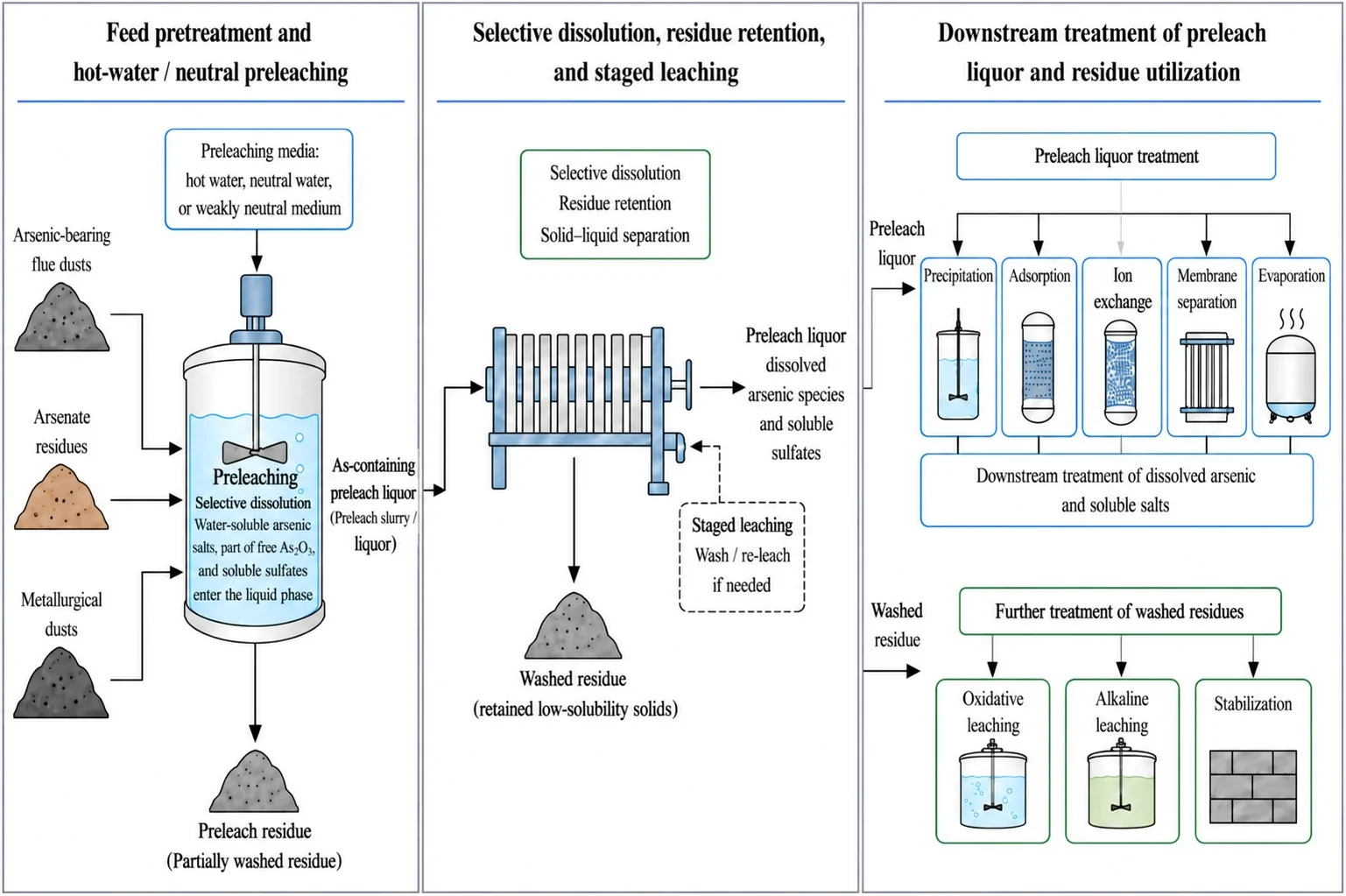
Schematic process flow for hot-water/neutral leaching and arsenic separation. AI was used for this figure, specifically GPT 5.5, mainly for logical sorting.

Hot-water/neutral leaching is a relatively mild hydrometallurgical route for arsenic-bearing flue dusts, roasted products, solid wastes, and materials containing soluble arsenic oxides. It uses the solubility of As_2_O_3_, As_2_O_5_, and certain arsenates in hot water or near-neutral media to transfer arsenic to solution as arsenious acid, arsenic acid, or arsenate, while sparingly soluble Fe-, Si-, and Ca-bearing components remain in the leach residue. In hot-water or neutral systems, arsenic oxides can dissolve through hydration; As(III) can also be oxidized to the more readily immobilized As(V) form by an added oxidant [69]. Major reactions are given in Table 9, and the process flow is shown in Figure 9 [70].

As summarized in Table 9, As_2_O_3_ and As_2_O_5_ hydrate and dissolve to form H_3_AsO_3_ and H_3_AsO_4_, respectively, transferring arsenic from solid to liquid. Soluble arsenites and arsenates can also dissolve directly. In the presence of oxidants such as H_2_O_2_, As(III) can be oxidized to As(V). Under near-neutral conditions, As(V) is mainly present as H_2_AsO_4_^−^ and HAsO_4_^2−^, which can subsequently be removed by precipitation, adsorption, or immobilization [70].

Hot-water or neutral leaching dissolves water-soluble arsenic species while retaining sparingly soluble solids for further treatment. Arsenic-bearing flue dusts, arsenate residues, and metallurgical dusts are leached with hot water, neutral water, or weakly neutral media so that water-soluble arsenic salts, part of the free As_2_O_3_, and soluble sulfates enter the liquid phase. Arsenic in solution may occur as H_3_AsO_3_, H_2_AsO_4_^−^, HAsO_4_^2−^, and related species; in alkaline systems, arsenites can further occur as AsO_2_^−^. The leach liquor may be treated by precipitation, adsorption, ion exchange, membrane separation, or evaporation, whereas washed residues can enter oxidative leaching, alkaline leaching, or stabilization operations.

#### 3.2.4. Inorganic-Salt Leaching

Inorganic-salt leaching uses salt media to control the dissolution and transfer behavior of arsenic. It can be applied to arsenic-bearing metallurgical dusts, sludges, arsenate slags, and complex arsenic-bearing solid wastes. Inorganic salts such as NaCl, NH_4_Cl, FeCl_3_, Fe_2_(SO_4_)_3_, Na_2_S, and Na_2_CO_3_ alter ionic strength, complexation, or redox conditions and thereby dissolve, transform, or selectively leach arsenic-bearing phases. Arsenic then enters solution as arsenite, arsenate, thioarsenate, or complexed ionic species, while some metal-bearing components and sparingly soluble impurities remain in the residue. Arsenic oxides, metal arsenates, and arsenic sulfides can dissolve, undergo ion exchange, or be oxidatively leached in these systems [71]. Major reactions are listed in Table 10.

**Figure 10:**
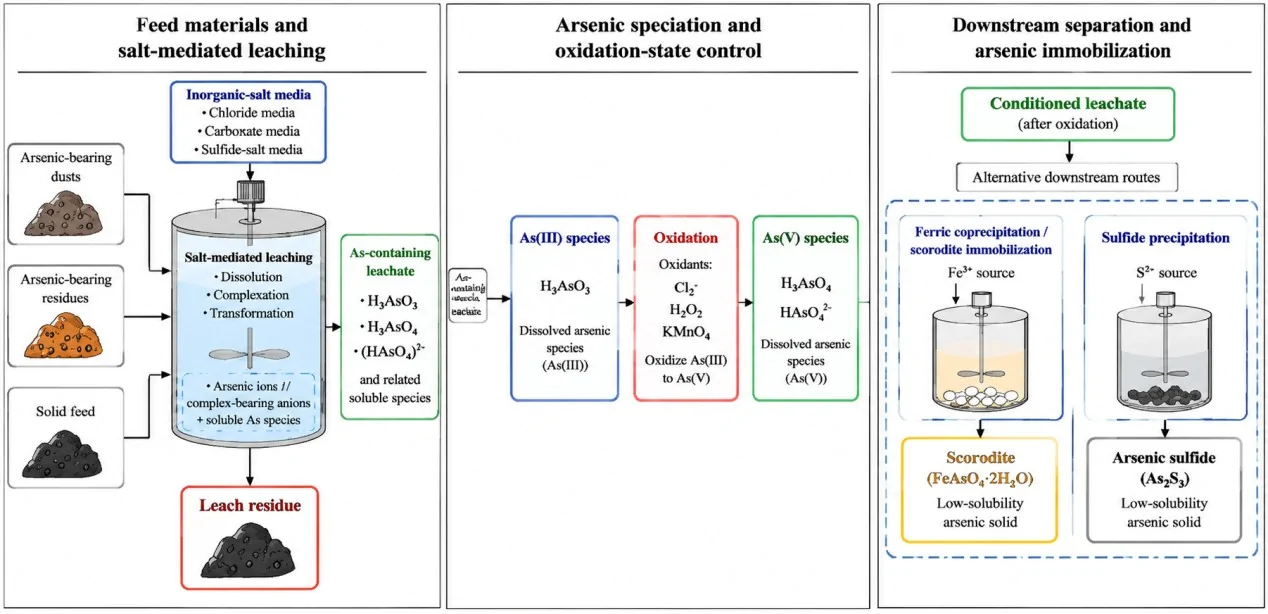
Schematic process flow for inorganic-salt leaching and arsenic separation. AI was used for this figure, specifically GPT 5.5, mainly for logical sorting.

As shown in Table 10, arsenic oxides and some metal arsenates can be converted to soluble arsenites or arsenates in carbonate media such as Na_2_CO_3_. Arsenic sulfides can form soluble thioarsenates in Na_2_S systems. Ferric salts such as FeCl_3_ oxidize As(III) to As(V) and assist oxidative leaching of arsenic sulfides, transferring arsenic selectively to the solution. A process schematic is shown in Figure 10 [72].

Inorganic-salt leaching combines salt-mediated dissolution with adjustment of the arsenic oxidation state and subsequent separation or immobilization [73]. Arsenic-bearing dusts and residues can be leached in chloride, carbonate, or sulfide-salt media, where arsenic oxides or arsenic-bearing minerals undergo dissolution, complexation, or transformation to yield soluble species such as H_3_AsO_3_, H_2_AsO_4_^−^, and HAsO_4_^2−^. Oxidants including Cl_2_, H_2_O_2_, and KMnO_4_ can convert As(III) to the more readily precipitated and immobilized As(V) form [74]. Depending on arsenic speciation and solution conditions, the leachate can then undergo ferric coprecipitation/scorodite immobilization or sulfide precipitation, converting arsenic into low-solubility FeAsO_4_·2H_2_O or As_2_S_3_ solids.

#### 3.2.5. Lime Neutralization and Precipitation

**Figure 11:**
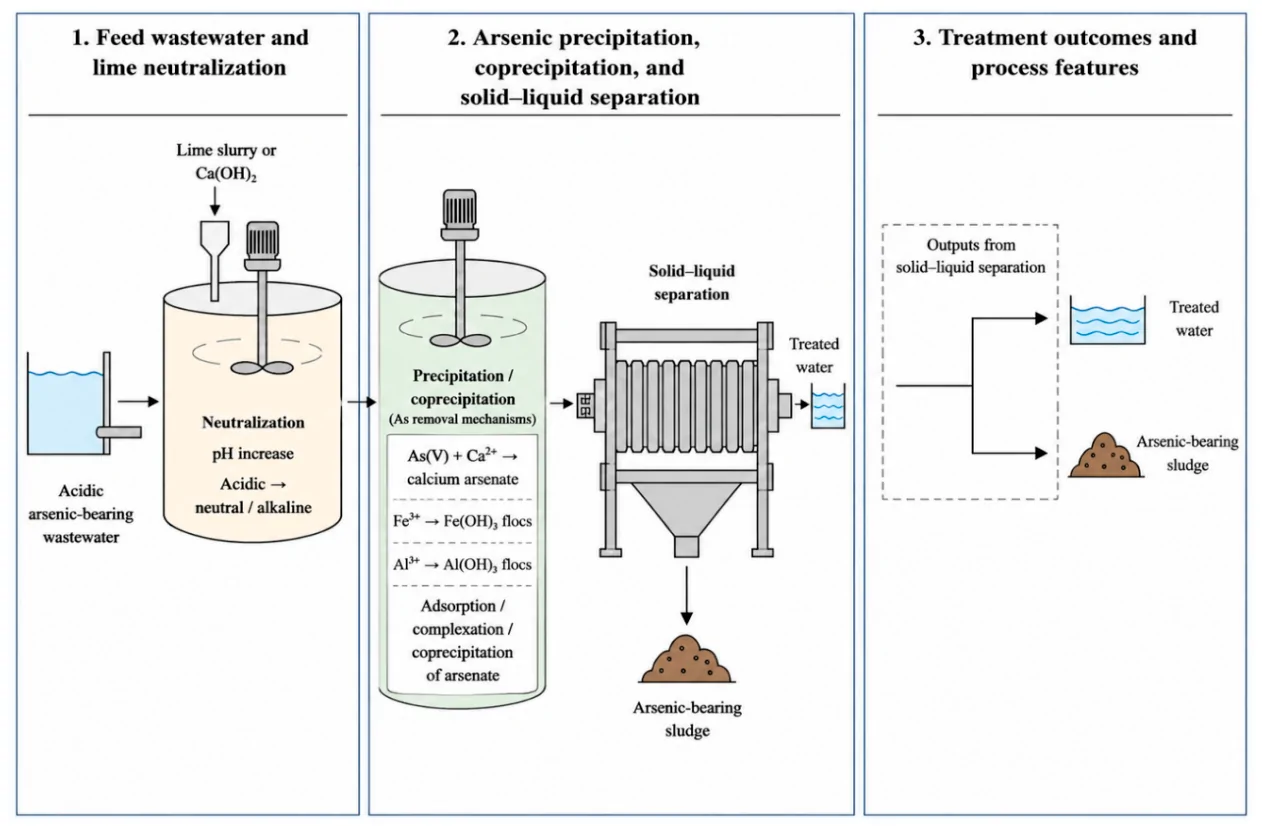
Schematic process flow for lime neutralization and precipitation of arsenic removal. AI was used for this figure, specifically GPT 5.5, mainly for logical sorting.

Lime neutralization and precipitation is a common wet-chemical method for treating arsenic-bearing acidic wastewater, metallurgical waste acid, leachates, and industrial effluents. Lime or lime slurry increases solution pH and promotes neutralization, precipitation, and coprecipitation of As(III) or As(V) with Ca^2+^ and OH^−^. The resulting calcium arsenate, calcium arsenite, or arsenic-bearing hydroxide precipitates transfer arsenic from liquid to solid. Under alkaline conditions, As(V) is more readily precipitated with Ca^2+^, while coexisting Fe and Al may form hydroxides that enhance arsenic removal through adsorption or coprecipitation [75,76]. Major reactions are listed in Table 11, and the process schematic is shown in Figure 11 [77].

As shown in Table 11, CaO first hydrates to Ca(OH)_2_, which releases Ca^2+^ and OH^−^ and neutralizes acidic arsenic-bearing solutions. As the pH rises, As(V) and As(III) can react with Ca^2+^ to form calcium arsenate and calcium arsenite precipitates, transferring arsenic to the solid phase. Coexisting Fe^3+^ can form colloidal Fe(OH)_3_, which also removes arsenic by adsorption or coprecipitation [77].

The process neutralizes acidic arsenic-bearing wastewater, precipitate or coprecipitate arsenic, and separates the resulting sludge. Lime slurry or Ca(OH)_2_ is added to raise the pH from the acidic range toward neutral or alkaline conditions, thereby creating conditions for precipitation [78]. As(V) can react with Ca^2+^ to form calcium arsenate, while coexisting Fe^3+^ and Al^3+^ can form Fe(OH)_3_ and Al(OH)_3_ colloidal flocs that remove arsenate by adsorption, complexation, or coprecipitation [79,80]. Solid–liquid separation produces treated water and arsenic-bearing sludge. The process is simple and comparatively low-cost, but it can generate large quantities of sludge. More importantly, calcium arsenate/calcium arsenite precipitates should not be regarded as intrinsically stable terminal residues. Their long-term behavior depends on precipitation pH, Ca/As ratio, mineral crystallinity, aging, and exposure to atmospheric CO_2_. Changes in pH and carbonation can induce phase transformation and arsenic remobilization. When lime precipitation is used for terminal arsenic stabilization, long-term leaching and aging tests are needed in addition to initial arsenic-removal measurements.

#### 3.2.6. Sulfide Precipitation

**Figure 12:**
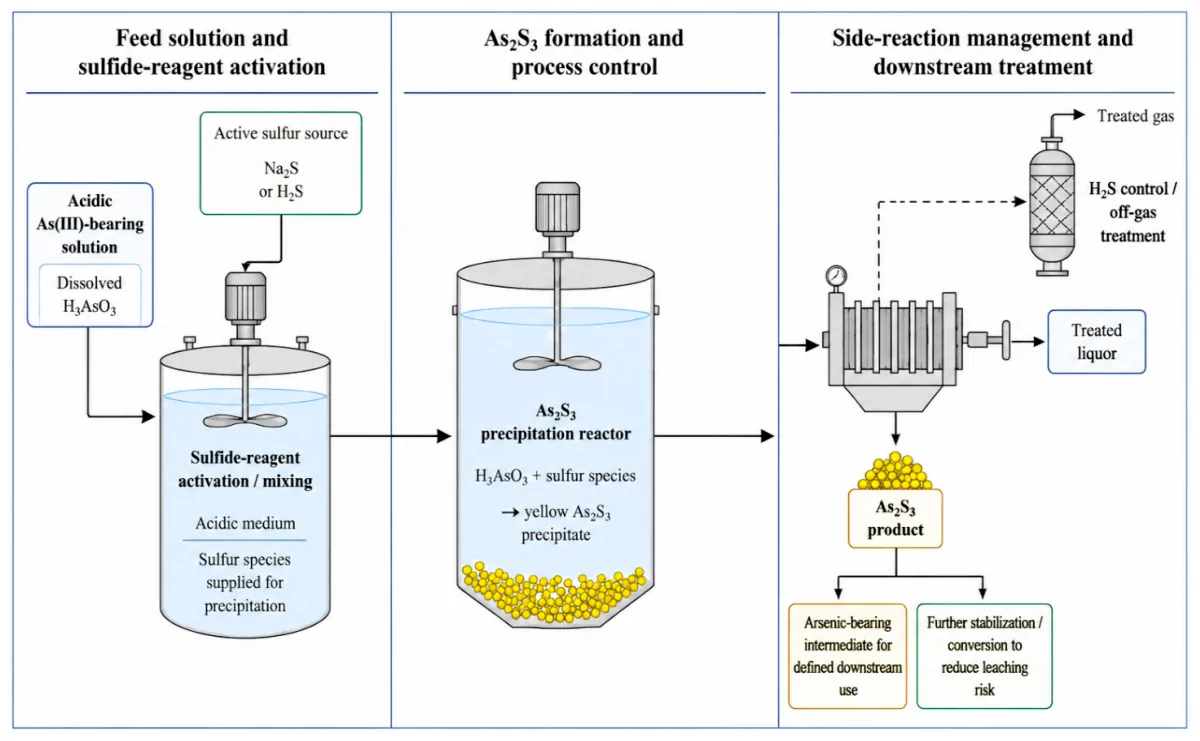
Schematic process flow for sulfide-precipitation arsenic removal. AI was used for this figure, specifically GPT 5.5, mainly for logical sorting.

Sulfide precipitation is a common wet-chemical route for arsenic-bearing acidic wastewater, metallurgical waste acid, leachates, and industrial effluents [81]. Sulfide reagents such as Na_2_S and H_2_S provide S^2−^ or HS^−^, which react with dissolved As(III) or As(V) to form sparingly soluble arsenic sulfides and transfer arsenic from liquid to solid. Under acidic conditions, As(III) more readily forms As_2_S_3_. As(V) usually requires prior reduction to As(III), or concomitant reduction during sulfidation, to improve precipitation efficiency and product stability [82,83]. Major reactions are listed in Table 12, and the process flow is shown in Figure 12 [84].

As summarized in Table 12, sulfide precipitation relies on H_2_S or S^2−^ released from sulfide reagents in acidic media. As(III) can react directly with H_2_S to form sparingly soluble As_2_S_3_. As(V) is commonly reduced during sulfidation and converted to As_2_S_3_, with elemental sulfur also produced. In thioarsenate systems, acidification can decompose thioarsenates and precipitate As_2_S_3_, thereby transferring arsenic from liquid to solid [85,86].

Sulfide precipitation converts dissolved arsenic to As_2_S_3_; reagent dosing and side reactions determine precipitate formation and downstream handling. Na_2_S or H_2_S provides the active sulfur source in acidic As(III)-bearing solutions [87]. Dissolved H_3_AsO_3_ reacts with sulfur species to form yellow As_2_S_3_ precipitate; precipitation is affected by pH, S/As molar ratio, Fe^3+^ concentration, seed addition, and solid–liquid separation conditions [88]. The precipitated As_2_S_3_ is an arsenic-rich intermediate, not an inherently stable disposal phase. Its dissolution and oxidation behavior is strongly dependent on pH, dissolved oxygen, and temperature. Arsenic release generally increases under more alkaline and oxidizing conditions, where As_2_S_3_ can progressively dissolve and arsenic may be converted from As(III) to dissolved As(V). Long-term storage or disposal of As_2_S_3_ must account for environmental redox conditions, pH variation, and any subsequent stabilization or controlled conversion.

#### 3.2.7. Ferric Coprecipitation (Lime–Ferric-Salt Process)

**Figure 13:**
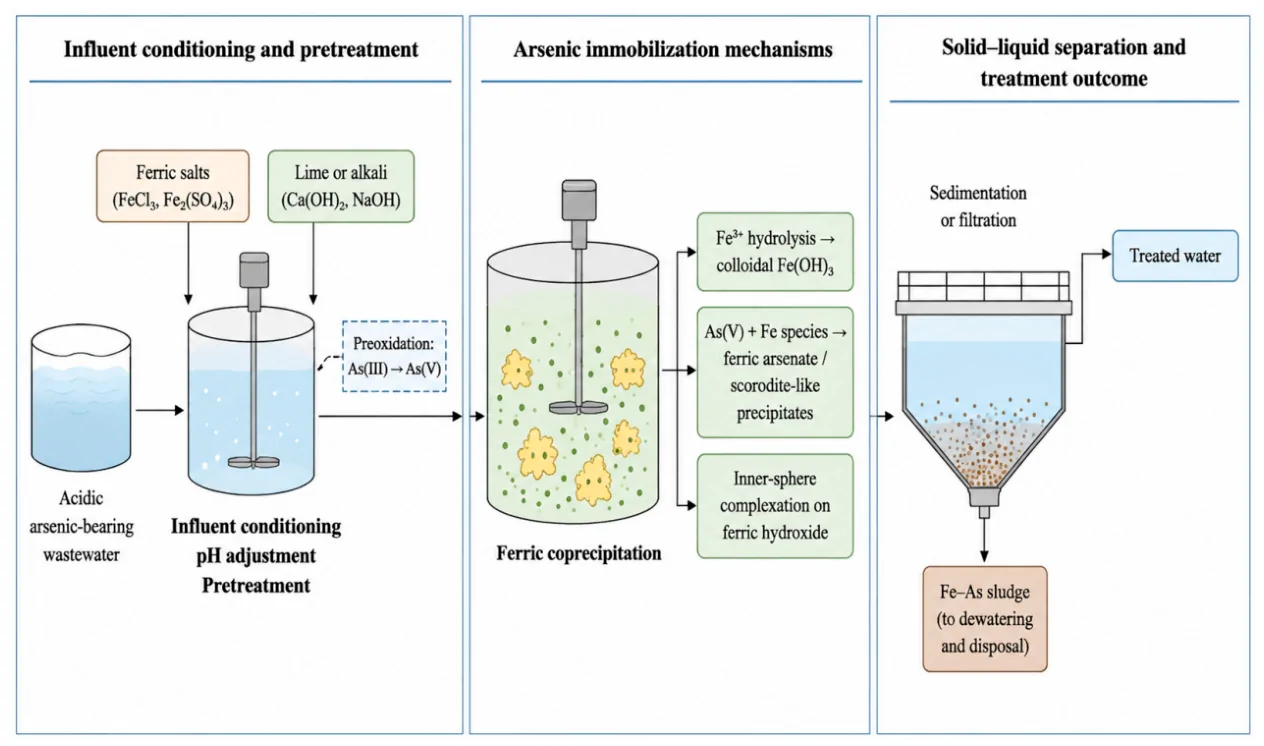
Schematic process flow for ferric coprecipitation (lime–ferric-salt) arsenic removal. AI was used for this figure, specifically GPT 5.5, mainly for logical sorting.

Ferric coprecipitation, often implemented as a lime–ferric-salt process, is commonly used for arsenic-bearing acidic wastewater, metallurgical waste acid, leachates, and industrial effluents. Ferric salt and lime slurry are added to the arsenic-bearing solution to adjust pH and hydrolyze Fe^3+^ into colloidal Fe(OH)_3_. As(V) can form ferric arsenate precipitates with Fe^3+^ or can be immobilized on ferric hydroxide through adsorption, complexation, and coprecipitation, transferring arsenic from liquid to solid. In lime–ferric-salt systems, As(III) is normally preoxidized to As(V) to improve arsenic removal and residue stability. Major reactions are listed in Table 13, and the process flow is shown in Figure 13 [89].

As shown in Table 13, lime hydration and neutralization increase solution pH and promote hydrolysis of Fe^3+^ to colloidal Fe(OH)_3_. Dissolved As(III) is commonly oxidized to As(V), which can form ferric arsenate with Fe^3+^ or be retained on ferric hydroxide flocs through surface complexation and adsorption. At higher pH, part of the arsenate can also precipitate with Ca^2+^ as calcium arsenate [89].

Ferric coprecipitation comprises influent conditioning and pretreatment, arsenic immobilization mechanisms, and solid–liquid separation. Ferric salts and lime or alkali are added to acidic arsenic-bearing wastewater. pH adjustment and pre-oxidation of As(III) to As(V) create conditions for subsequent arsenic precipitation [90]. Fe^3+^ hydrolyzes to colloidal Fe(OH)_3_, while As(V) can form ferric arsenate or scorodite-like precipitates and can be retained on ferric hydroxide by inner-sphere complexation. Sedimentation or filtration then produces treated water and Fe–As sludge. Although ferric coprecipitation is an established approach for arsenic removal from wastewater, the resulting Fe–As sludge is generally heterogeneous and may contain arsenate adsorbed on ferrihydrite, poorly crystalline ferric arsenate, and other Fe-bearing phases rather than a single well-defined crystalline product. Its long-term stability therefore depends on the Fe/As ratio, pH, crystallinity, aging, and environmental conditions. Aging-induced transformation of poorly crystalline Fe phases can alter arsenic retention and may promote arsenic release. Its widespread use in wastewater treatment does not, by itself, demonstrate long-term stability of the resulting Fe–As sludge [91].

#### 3.2.8. Scorodite Crystallization

**Figure 14:**
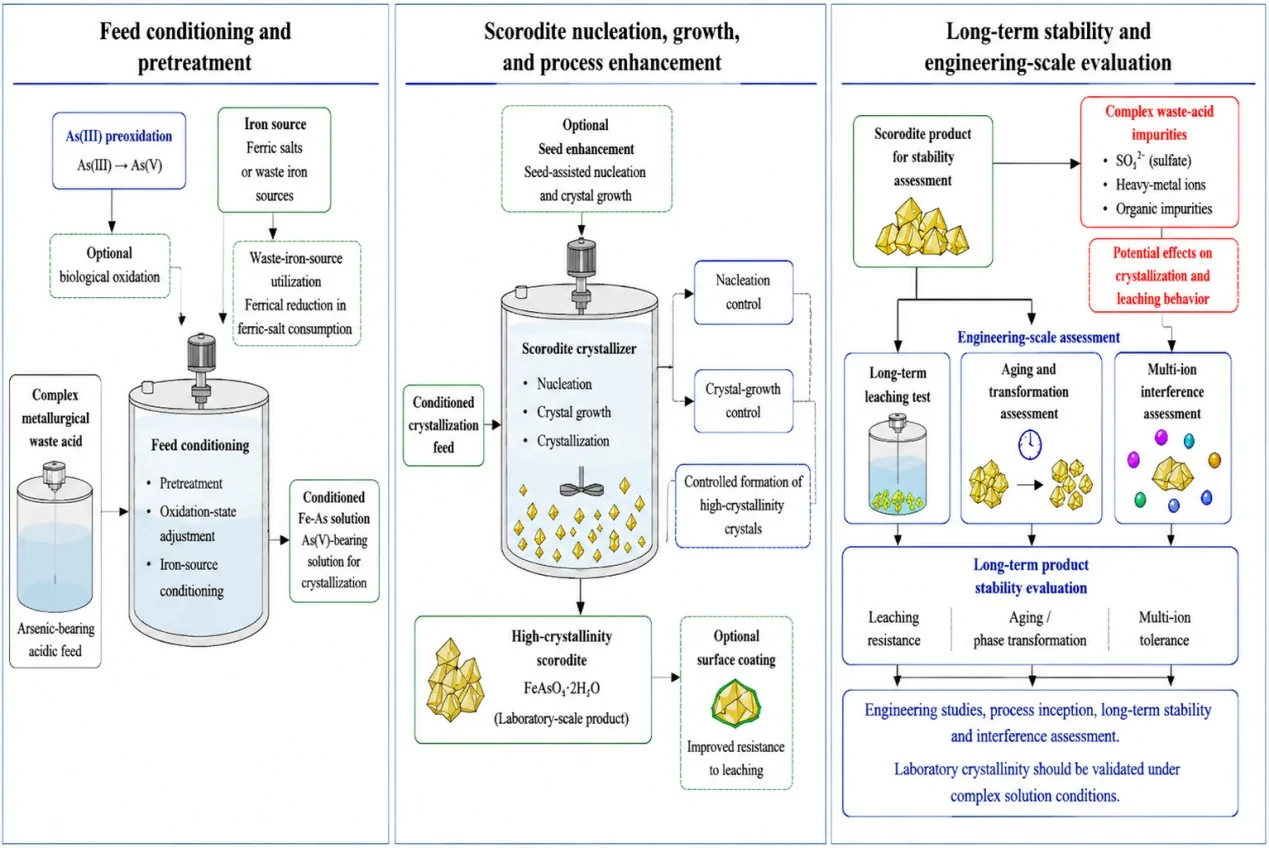
Schematic process flow for scorodite crystallization–arsenic removal. AI was used for this figure, specifically GPT 5.5, mainly for logical sorting.

Scorodite crystallization is a wet-chemical arsenic immobilization route based on the formation of crystalline FeAsO_4_·2H_2_O from Fe(III) and As(V). Compared with amorphous Fe–As precipitates and calcium arsenate sludge, well-crystallized scorodite can exhibit relatively low arsenic leachability, but only under specified synthesis and exposure conditions [92,93]. Scorodite generally exhibits higher stability under mildly acidic and oxidizing conditions, whereas arsenic release can increase under strongly acidic, neutral-to-alkaline, or reducing environments. Its long-term behavior also depends on crystallinity, synthesis pH, Fe/As molar ratio, temperature, redox potential, seed addition, sulfate incorporation, and coexisting impurity ions [94]. Reports of scorodite stability should specify both synthesis conditions and subsequent environmental exposure instead of describing scorodite simply as a “stable arsenic-bearing phase.” Major reactions are given in Table 14, and the process flow is shown in Figure 14 [95].

As summarized in Table 14, the process relies on crystallization of As(V) with Fe(III). As(III) must usually be oxidized to As(V), and Fe(II) can be oxidized to Fe(III); Fe^3+^ then reacts with H_3_AsO_4_ or H_2_AsO_4_^−^ to precipitate low-solubility FeAsO_4_·2H_2_O [96,97]. At excessively high pH, Fe^3+^ hydrolyzes to Fe(OH)_3_, consuming ferric salt and inhibiting scorodite formation. pH, Fe/As molar ratio, and redox conditions must therefore be controlled [98,99].

Scorodite crystallization requires feed conditioning, control of nucleation and crystal growth, and verification of long-term product stability [96]. Recent studies have explored biological oxidation, use of waste iron sources, seed enhancement, and surface coatings to reduce ferric-salt consumption and improve resistance to leaching [100,101]. However, high-crystallinity products obtained in laboratory systems can still be affected by sulfate, heavy-metal ions, and organic impurities in complex metallurgical waste acids [96,102]. Scale-up studies should include long-term leaching and aging tests and quantify interference from coexisting ions [103,104].

#### 3.2.9. Ion Exchange and Adsorption

**Figure 15:**
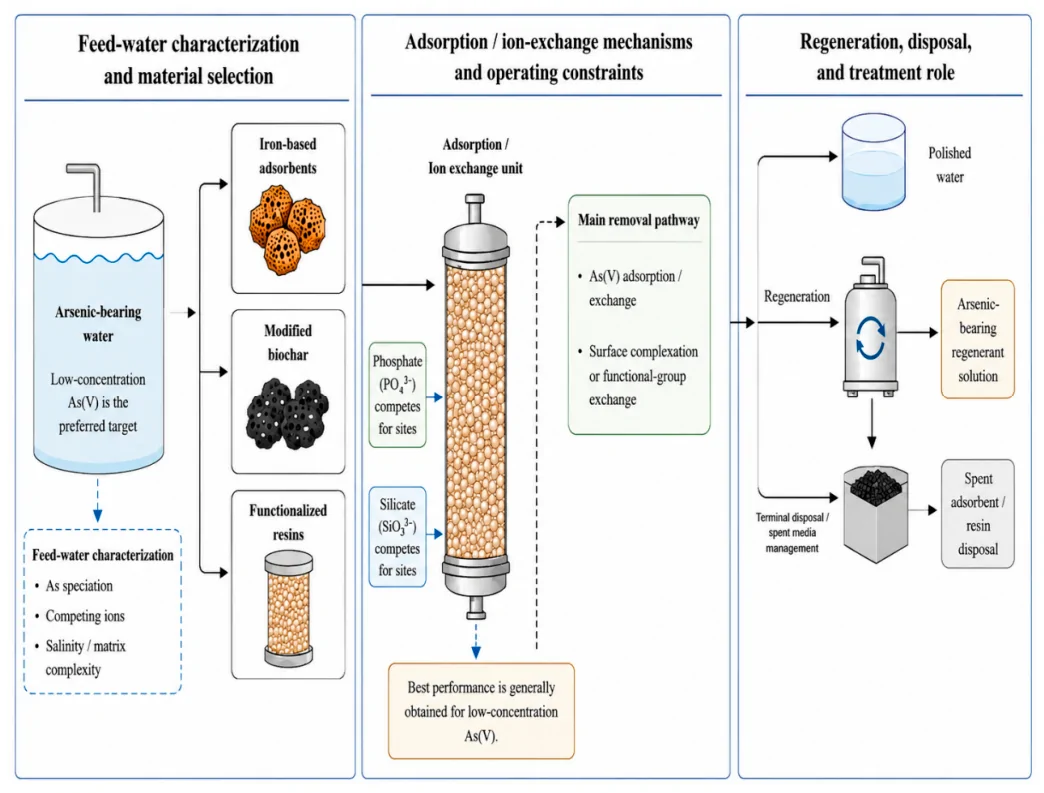
Schematic process flow for ion exchange and adsorption in arsenic removal. AI was used for this figure, specifically GPT 5.5, mainly for logical sorting.

Ion exchange and adsorption are commonly used for deep arsenic removal from arsenic-bearing groundwater, industrial wastewater, purified metallurgical waste acid, and low-concentration arsenic-bearing leachates [105]. The methods use active sites on ion-exchange resins, activated alumina, iron oxides, iron–manganese oxides, modified biochars, zeolites, and related materials to selectively exchange, complex, or adsorb As(III) and As(V), transferring arsenic from liquid to the sorbent surface [106,107]. As(V), generally present as H_2_AsO_4_^−^ or HAsO_4_^2−^, is more readily adsorbed or exchanged, whereas As(III) often occurs as neutral H_3_AsO_3_ and is less readily removed. Practical treatment therefore often preoxidizes As(III) to As(V) [108,109]. Major reactions are listed in Table 15, and the process flow is shown in Figure 15.

As shown in Table 15, ion exchange and adsorption depend on arsenic oxidation state, aqueous speciation, and interactions with solid-surface active sites. As(III) can be oxidized to As(V), which occurs in solution principally as H_2_AsO_4_^−^ and HAsO_4_^2−^ and can then be retained on iron oxides or alumina through surface complexation, electrostatic adsorption, or ligand exchange [110]. Anion-exchange resins can capture arsenate by exchanging Cl^−^, OH^−^, and other exchangeable anions [111].

Application of ion exchange or adsorption requires characterization of the feed water, selection of the sorbent, and a plan for regeneration or disposal [112,113]. Iron-based adsorbents, modified biochar, and functionalized resins can remove low-concentration As(V), but neutral As(III) and competing phosphate or silicate limit performance, and arsenic-bearing regenerant solutions still require treatment [110,111,112,113,114,115,116,117] .

#### 3.2.10. Solvent Extraction

**Figure 16:**
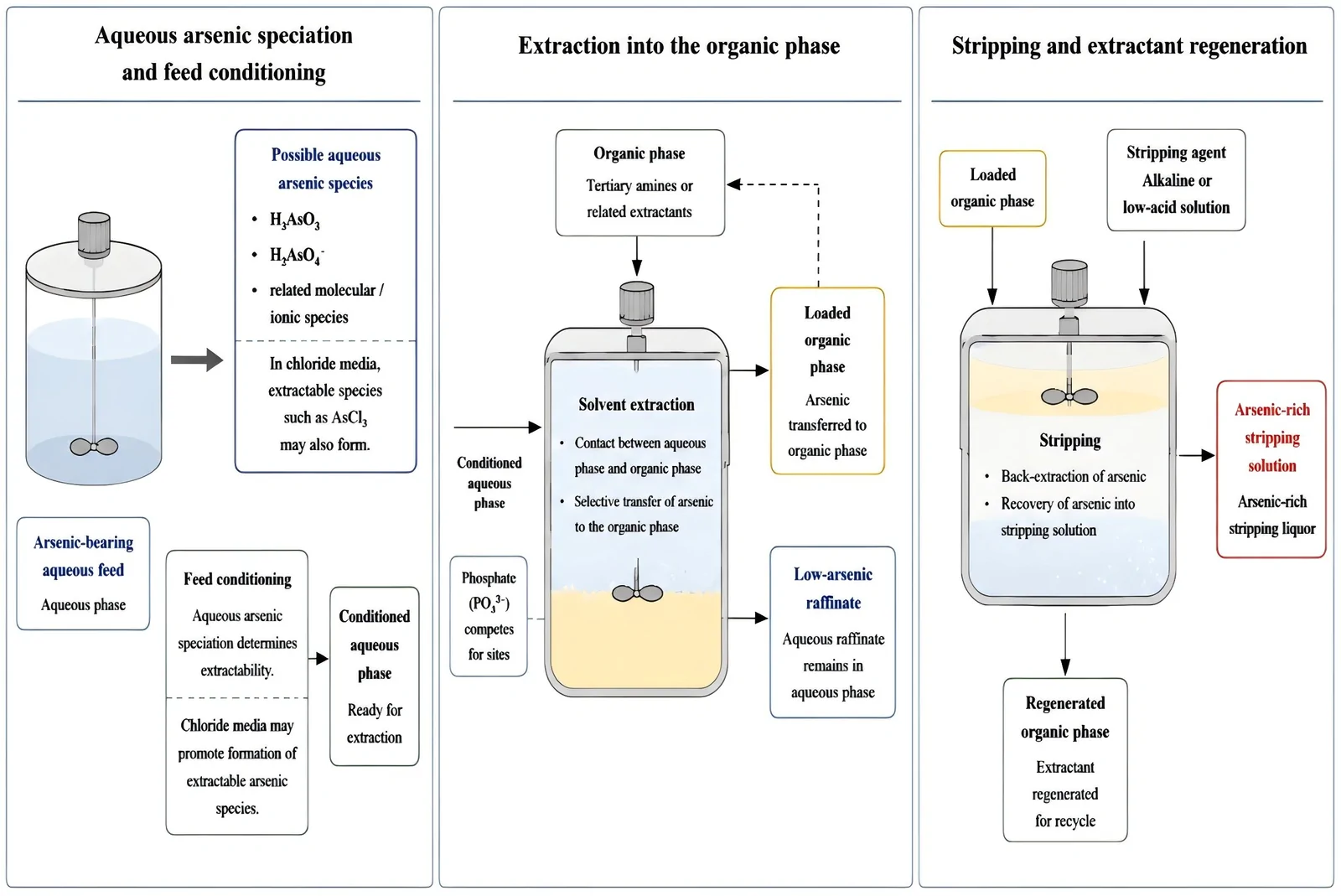
Schematic process flow for solvent extraction arsenic removal. AI was used for this figure, specifically GPT 5.5, mainly for logical sorting.

Solvent extraction separates arsenic and associated metals by exploiting differences in their distribution between aqueous and organic phases [114,115]. It can be used for arsenic-bearing leachates, metallurgical waste acid, industrial effluents, and complex polymetallic solutions. Appropriate selection of extractant, diluent, and stripping solution allows arsenic to transfer selectively to the organic phase as neutral molecules, complexes, or ion pairs, while copper, zinc, lead, iron, and other coexisting metals remain in the aqueous phase or report to a different separation stream. Under suitable pH, acidity, and phase-ratio conditions, As(V) or As(III) complexes, associates, or solvates with organic extractants and is separated selectively from coexisting components [118,119]. Major extraction reactions or equilibria are listed in Table 16, and the process flow is shown in Figure 16 [120].

As summarized in Table 16, solvent extraction relies on complexation, ion association, and solvation equilibria between arsenic species in the aqueous and organic phases. In chloride media, As(III) can form neutral AsCl_3_ and undergo solvating extraction with phosphate-ester or phosphine-oxide extractants. As(V) typically occurs as H_2_AsO_4_^−^ or HAsO_4_^2−^ and can transfer to a protonated amine extractant by anion exchange [121,122]. Subsequent stripping with alkaline solution, dilute acid, or water returns arsenic to the aqueous phase and regenerates the extractant.

Solvent extraction requires adjustment of aqueous arsenic speciation, transfer to the organic phase, and subsequent stripping and extractant regeneration. In the aqueous phase, arsenic may occur as H_3_AsO_4_, H_2_AsO_4_^−^, and related molecular or ionic species; in chloride media, extractable species such as AsCl_3_ may also form. Contact between the aqueous phase and an organic phase containing tertiary amines or related extractants allows arsenate to form ion pairs or undergo anion exchange, transferring arsenic selectively to the organic phase while low-arsenic raffinate remains aqueous [123,124]. The loaded organic phase is stripped with alkaline or low-acid solution to yield an arsenic-rich stripping solution and regenerate the extractant for recycling.

#### 3.2.11. Comparison of Hydrometallurgical Separation Technologies

Data are listed in Table 17.

**Table 17:** Comparison of hydrometallurgical arsenic separation technologies.

Process	Representative Feedstock	Representative Reagent/pH Conditions	Temperature	Time	As Separation/Removal Performance	Valuable Metal Recovery/Selectivity	Final As Form	Development Status	Process
Acidic pressure leaching	Copper-smelting flue dust	H_2_SO_4_ 30 g L^−1^; O_2_ partial pressure 0.6 MPa; Fe/As = 1.5; L/S = 5:1 [124]	160 °C	180 min	95.86% As precipitation during leaching	98.95% Cu leaching	Scorodite-rich arsenic-bearing precipitate	Laboratory-scale; industrial metallurgical flue dust	Acidic pressure leaching
Ultrasound-assisted alkaline leaching	Copper-smelting flue dust	0.4 M NaOH + 0.2 M Na_2_S; L/S = 50 mL g^−1^; ultrasound 150 W, 100 kHz	80 °C	45 min	99% As extraction [125]	Extraction of Cu, Zn and Pb < 1%	Arsenic-rich alkaline leachate	Laboratory-scale	Ultrasound-assisted alkaline leaching
Hot-water leaching after acid roasting	Gold concentrate/acid-baked product	H_2_SO_4_ roasting pretreatment; subsequent water leaching; representative water-leach S/L ≈ 2 g/100 mL	Roasting 400 °C; water leach 70 °C	Roasting 4 h; water leach 60 min	81.43% As leaching; 92.03% As removal from solid residue	Fe leaching 80.02%; Cu 70.10%, Zn 81.93%, Pb 50.48% under corresponding maximum conditions; Au/Ag enriched in residue	Arsenic-bearing aqueous leachate	Laboratory-scale	Hot-water leaching after acid roasting
Hypochlorite inorganic-salt leaching	Enargite-bearing Cu concentrate	NaClO/ClO^−^ 0.1–0.3 M; pH 12–12.5	20–40 °C	<60 min	Feed As decreased from >0.5 wt% to <0.2 wt% or 0.2–0.5 wt%	Enargite/chalcopyrite selectivity factor ≈ 5:1; Cu and Fe mainly retained as CuO and Fe(OH)_3_	Dissolved arsenate/arsenite species	Laboratory-scale	Hypochlorite inorganic-salt leaching
Lime neutralization–compound-salt precipitation	As-containing acidic smelting wastewater	Lime neutralization at pH 3; compound precipitation at pH 8.5; Cu/As = 0.45–2.3, Fe/As = 1.20–6.0, Zn/As = 1.20–6.0	Room temperature for lime neutralization	0.5 h neutralization; subsequent step process-dependent	Final dissolved As <0.5 mg L^−1^; downstream As_2_O_3_ recovery 96.5%	Metals incorporated/recycled through compound salt system; no independent metal-recovery percentage reported	CaSO_4_-rich residue + recoverable As_2_O_3_	Laboratory-scale; engineering-oriented process	Lime neutralization–compound-salt precipitation
Controlled H_2_S sulfide precipitation	Strongly acidic arsenic-bearing wastewater	H_2_S supplied through PTFE membrane contactor; controlled bubble-free H_2_S transfer	NR	NR	99.3% As precipitation [87]	Cu/As separation factor up to 7490.9	As_2_S_3_-rich sulfide precipitate	Laboratory-scale	Controlled H_2_S sulfide precipitation
Lime–ferric-salt coprecipitation	Copper-smelting waste acid; initial As 10,034 mg L^−1^	Lime to pH 5.5; FeSO_4_ + H_2_O_2_; Fe/As = 1.05	70 °C	NR	As decreased from 10,034 to 900 mg L^−1^; 81.4% removal in ferric/lime stage	N/A	Fe–As sludge/ferric arsenate-bearing precipitate	Laboratory-scale	Lime–ferric-salt coprecipitation
Scorodite crystallization	Hydrometallurgical waste sulfuric acid	Initial pH 1.1; Fe/As = 2; siderite as Fe source	95 °C	10 h	99.99% As removal [126]	N/A	Scorodite/ferric arsenate residue; TCLP As 0.13 mg L^−1^	Laboratory-scale using real WSA	Scorodite crystallization
Co-loaded resin adsorption/ion exchange	As(V)-containing wastewater; initial As 100 mg L^−1^	Optimum pH 8	NR/ambient-type batch condition	0–30 min for >99% removal	>99% As(V) removal [111]	N/A; adsorption capacity up to 116 mg g^−1^	Loaded co-resin	Laboratory-scale	Co-loaded resin adsorption/ion exchange
Cyanex 923 solvent extraction	As(V)-containing H_2_SO_4_ solution	As(V) 1.5 g L^−1^; H_2_SO_4_ 1.5 M; Cyanex 923 50 vol%; O/A = 1	20 °C	5 min	54% As extraction at 50 vol% Cyanex 923; up to 71% with neat Cyanex 923 [118]	At 1.5 M H_2_SO_4_: Cu extraction 5%, Bi ≈ 0%, Sb 26%, Ni ≈ 0%	Loaded organic → arsenic-rich strip liquor	Laboratory-scale	Cyanex 923 solvent extraction

### 3.3. Integrated Pyrometallurgical–Hydrometallurgical Separation Technologies

Integrated pyrometallurgical–hydrometallurgical separation couples thermal pretreatment with leaching, purification, or electrowinning. Thermal treatment first changes arsenic occurrence or concentrates arsenic through roasting, smelting, vacuum distillation, or high-temperature filtration. Leaching, selective precipitation, extraction, or electrowinning then separates arsenic more completely from valuable metals. Combined flowsheets can treat complex polymetallic arsenic-bearing feedstocks that are poorly suited to a single pyrometallurgical or hydrometallurgical process. Process control, energy consumption, stabilization of secondary slags and liquors, and continuous closed operation remain the principal constraints on industrial scale-up [127,128].

#### 3.3.1. Roasting–Leaching Process

**Figure 17:**
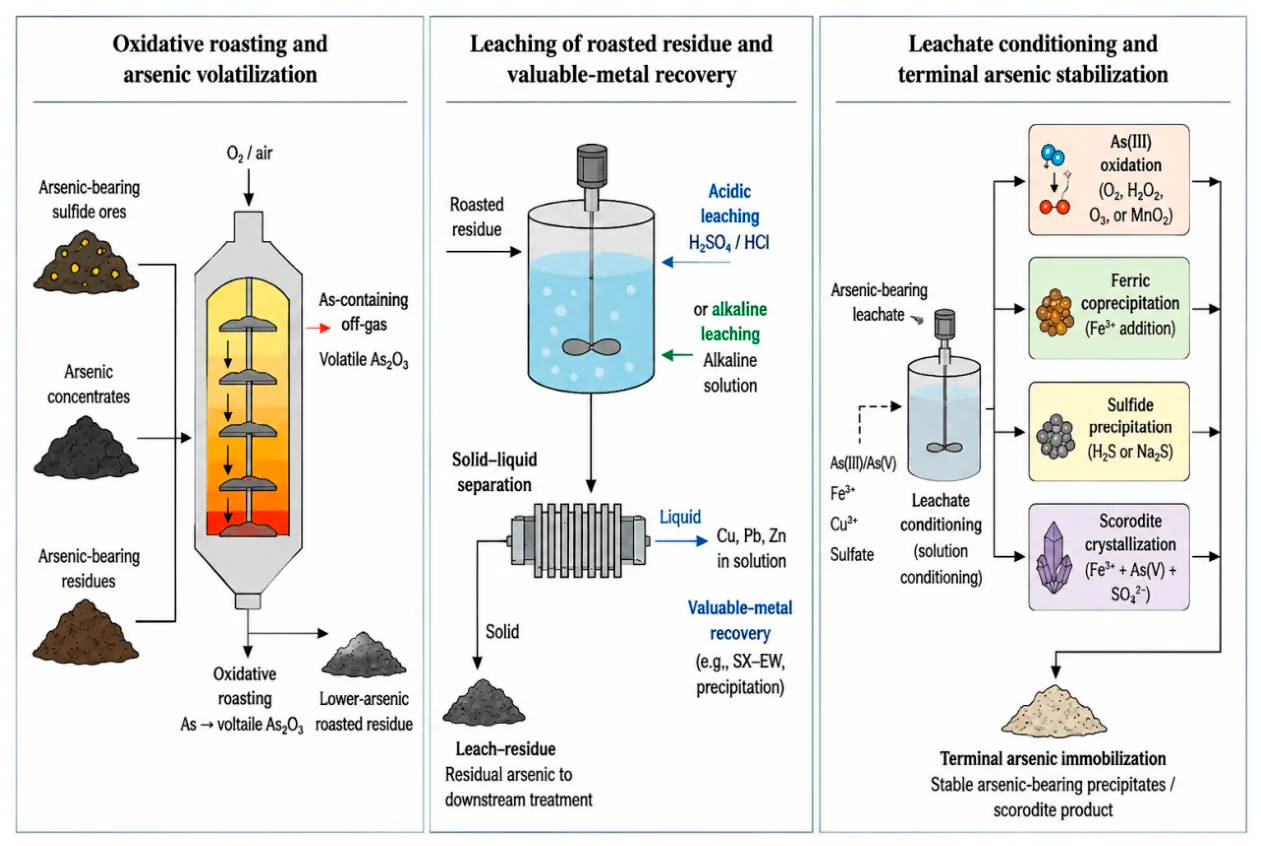
Schematic process flow for the roasting–leaching process. AI was used for this figure, specifically GPT 5.5, mainly for logical sorting.

The roasting–leaching process is a representative integrated route for arsenic-bearing sulfide ores, metallurgical flue dusts, arsenic-bearing slags, and complex polymetallic arsenic-bearing solid wastes. Oxidative or reduction roasting first alters the occurrence of arsenic, converting arsenic sulfides, arsenates, or complex arsenic-bearing phases into compounds that are more volatile, more leachable, or otherwise more separable. Subsequent acidic, alkaline, or salt-mediated leaching transfers arsenic selectively to solution or retains it in a stable slag/residue phase, thereby separating arsenic from copper, iron, and other components [129,130]. During roasting, arsenic can be converted to As_2_O_3_ and volatilize into flue dust. During leaching, residual arsenic or arsenic-enriched phases can be dissolved, transformed, or precipitated/immobilized. Major reactions are listed in Table 18, and the process flow is shown in Figure 17.

As shown in Table 18, oxidative roasting first converts arsenic sulfides or arsenopyrite to As_2_O_3_, which enters the off-gas as species such as As_4_O_6(g)_ and recondenses as solid As_2_O_3_ during cooling and dust collection. Residual As_2_O_3_ or arsenate phases in roasted residue can then enter solution through hydration or acidic leaching. As(III) in the leachate can subsequently be oxidized to As(V) before precipitation, crystallization, or stabilization, completing the thermal and wet separation sequence.

The roasting–leaching process includes arsenic volatilization during roasting, leaching of the roasted residue with valuable metal recovery, and leachate conditioning followed by terminal stabilization. Arsenic-bearing sulfide ores or concentrates undergo oxidative roasting so that arsenic converts to volatile As_2_O_3_. Cooling, dust collection, and condensation enrich the volatile product as crude As_2_O_3_ while producing a lower-arsenic roasted residue. Subsequent acidic or alkaline leaching of the residue transfers valuable metals such as Cu, Pb, and Zn to the solution for recovery, while residual arsenic proceeds with the leachate or residue to downstream treatment [131]. Depending on the solution chemistry, including As(III)/As(V), Fe^3+^, Cu^2+^, and sulfate concentrations, As(III) oxidation, ferric coprecipitation, sulfide precipitation, or scorodite crystallization can provide terminal arsenic immobilization.

#### 3.3.2. Vacuum-Distillation–Leaching Process

**Figure 18:**
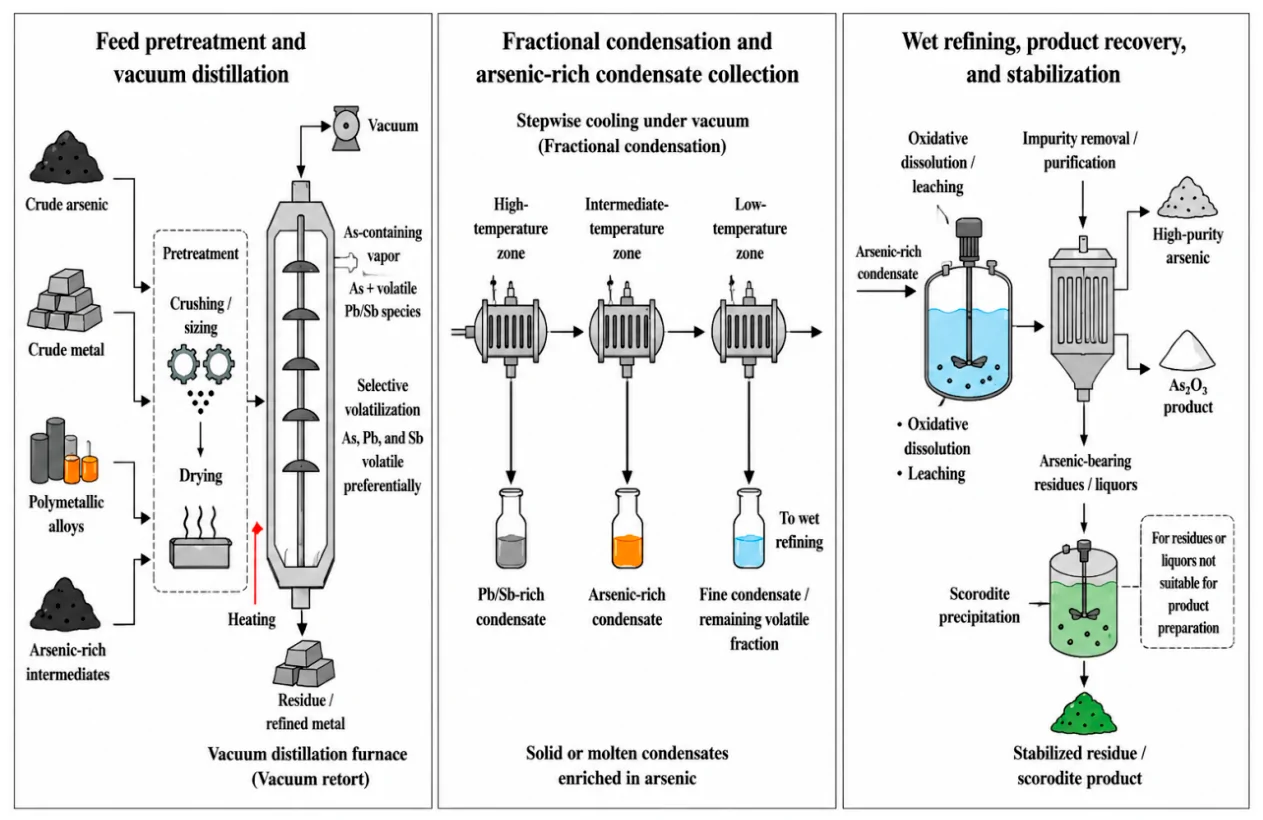
Schematic process flow for the vacuum distillation–leaching process. AI was used for this figure, specifically GPT 5.5, mainly for logical sorting.

The vacuum-distillation–leaching process couples vacuum volatilization with wet leaching for arsenic-bearing metallurgical flue dusts, alloys, arsenic-rich intermediates, and complex polymetallic solid wastes. Under vacuum or reduced pressure, differences in vapor pressure, boiling point, and sublimation temperature allow arsenic and its compounds to enter the gas phase as As_2_O_3_, As_4_O_6_, or elemental arsenic and to become enriched in the condensation zone. Acidic, alkaline, or salt-mediated leaching then transfers residual arsenic or valuable metals from the distillation residue or condensate into the solution for further separation from copper, lead, zinc, tin, and related components. Vacuum distillation preferentially volatilizes and enriches arsenic, whereas leaching cleans the residue, recovers valuable metals, or further purifies arsenic products [20,132]. Major reactions are given in Table 19, and the process flow is shown in Figure 18.

As summarized in Table 19, reduced pressure promotes preferential volatilization of As_2_O_3_ or elemental arsenic, which can condense as solid As_2_O_3_ or elemental arsenic in the collection zone. Condensed As_2_O_3_ can be hydrated and leached, followed by oxidation of As(III) to As(V) where precipitation or stabilization is needed. In the distillation residue, CuO and ZnO can be leached with sulfuric acid to form soluble sulfates, coupling arsenic enrichment with hydrometallurgical recovery of valuable metals [20,132].

In this flowsheet, selective vacuum volatilization is followed by fractional condensation and wet refining of the arsenic-rich fractions. Crude arsenic, crude metal, polymetallic alloys, or arsenic-rich intermediates are heated under vacuum, allowing arsenic and volatile impurities such as Pb and Sb to enter the gas phase preferentially while less volatile metals remain in the residue or refined metal. Stepwise cooling through different temperature zones produces solid or molten condensates enriched in arsenic for subsequent wet treatment [133,134]. Oxidative dissolution, leaching, impurity removal, and purification of arsenic-rich condensates can produce high-purity arsenic or As_2_O_3_ products. Arsenic-bearing residues or liquors that are not suitable for defined product preparation should be stabilized: for example, by scorodite crystallization.

#### 3.3.3. Smelting–Leaching–Electrowinning Process

**Figure 19:**
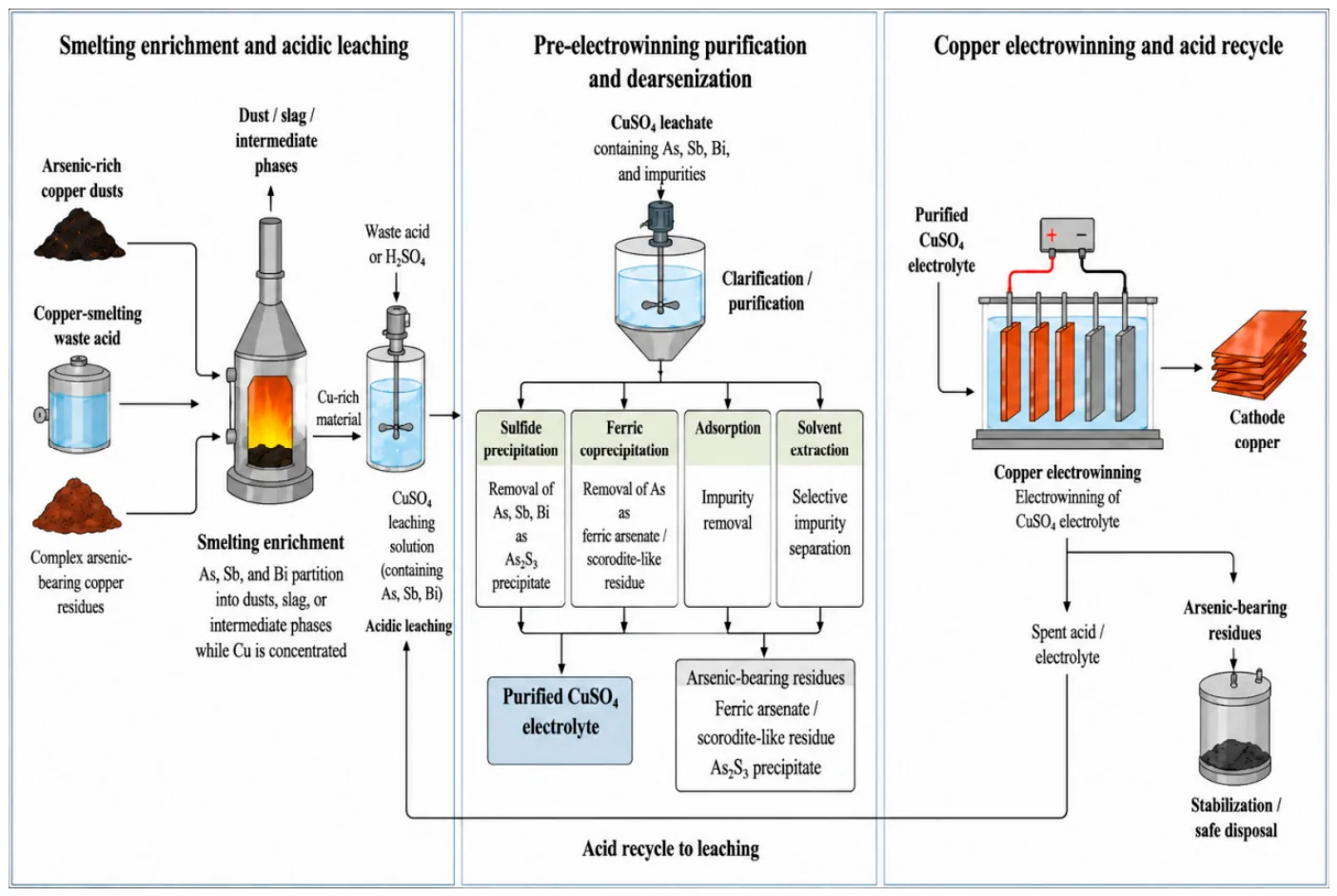
Schematic process flow for the smelting–leaching–electrowinning process. AI was used for this figure, specifically GPT 5.5, mainly for logical sorting.

The smelting–leaching–electrowinning process is a representative integrated route for arsenic-bearing copper concentrates, copper-smelting flue dusts, copper-smelting slags, and complex polymetallic intermediates. High-temperature smelting first partitions arsenic among the gas, slag, and metal or matte phases. Part of the arsenic volatilizes into dust or becomes immobilized in slag, while copper and other valuable metals become enriched in matte, blister copper, or Cu-rich intermediates. Acidic leaching subsequently transfers copper to solution; purification removes As, Sb, Bi, Fe, and other impurities; and the purified copper solution is sent to electrowinning to produce cathode copper. Off-gas dust collection, slag immobilization, or leachate purification directs arsenic away from the copper-recovery stream [135,136]. Major reactions are listed in Table 20, and the process flow is shown in Figure 19.

As shown in Table 20, high-temperature smelting oxidizes copper sulfides and transfers iron to slag through slag-forming reactions. Arsenic can be oxidized to As_2_O_3_ and volatilize as As_4_O_6(g)_, or it can form ferric arsenate in slag under stronger oxidation and an appropriate slag composition. Cu-rich oxides or intermediates are then leached with sulfuric acid to form CuSO_4_. As(III) in the leachate can be oxidized to As(V) and removed by ferric arsenate precipitation before the purified copper sulfate solution undergoes electrowinning to produce cathode copper [137].

This flowsheet combines smelting enrichment and acidic leaching with pre-electrowinning purification and copper electrowinning; the regenerated acid is returned to leaching [137]. Arsenic-rich copper-smelting flue dusts, copper-smelting waste acid, and complex arsenic-bearing copper residues are thermally enriched so that arsenic, antimony, and bismuth partition into dust, slag, or intermediate phases while copper is concentrated. Cu-rich material is then leached in waste acid or sulfuric acid, producing CuSO_4_ solution together with dissolved arsenic and impurities [138]. Before electrowinning, the leachate is clarified and purified through sulfide precipitation, ferric coprecipitation, adsorption, or solvent extraction to remove As, Sb, Bi, and related impurities, producing ferric arsenate, scorodite-like residues, or As_2_S_3_ precipitate [139]. The purified CuSO_4_ electrolyte is electrowon to cathode copper, acid is returned to leaching, and arsenic-bearing residues are sent to stabilization or final disposal.

#### 3.3.4. High-Temperature-Filtration–Hydrometallurgical Process

**Figure 20:**
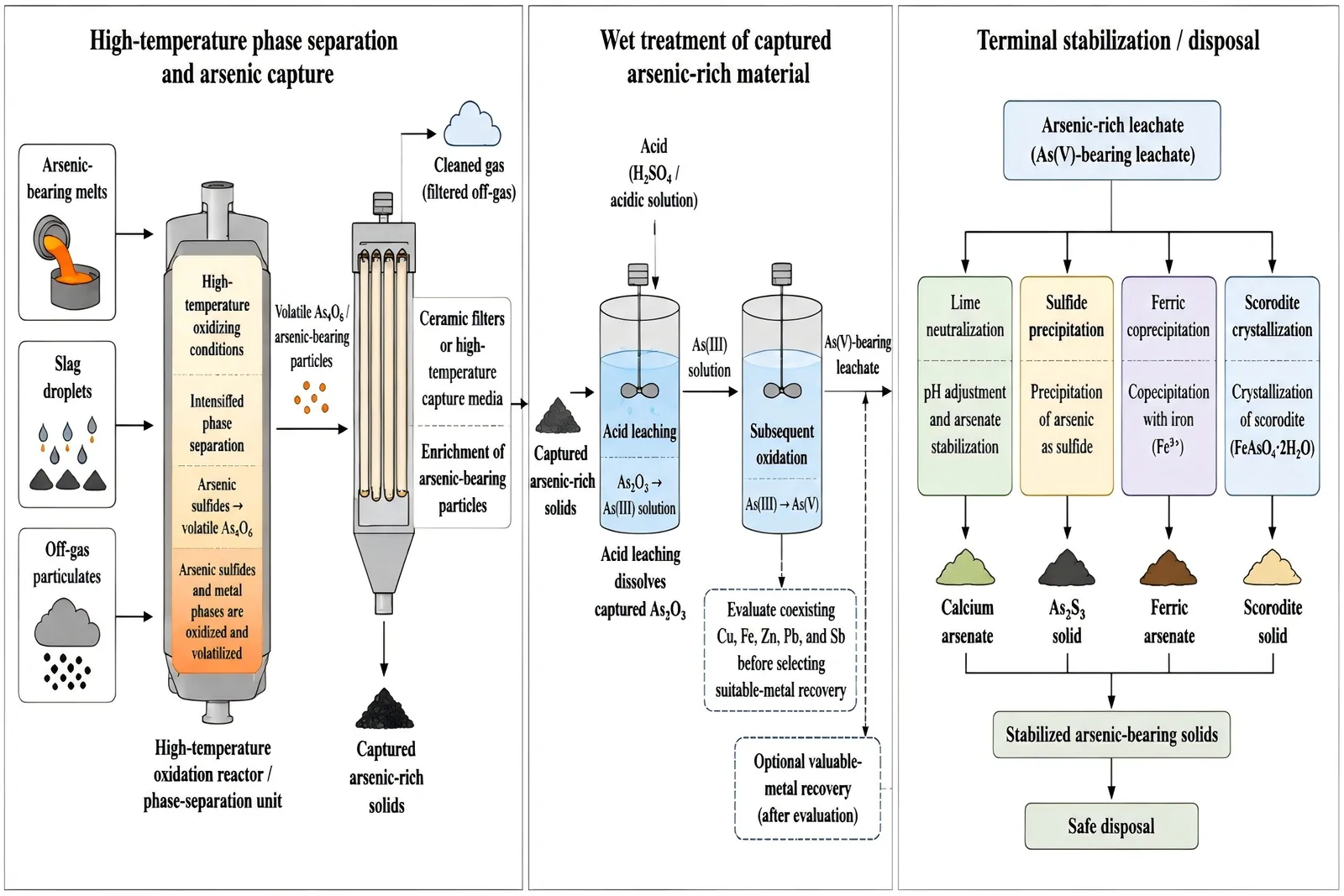
Schematic process flow for the high-temperature filtration–hydrometallurgical process. AI was used for this figure, specifically GPT 5.5, mainly for logical sorting.

The high-temperature-filtration–hydrometallurgical process couples high-temperature gas–solid separation with leaching, purification, or arsenic precipitation. It is applicable to arsenic-bearing metallurgical off-gases, flue dusts, volatile arsenic-bearing intermediates, and complex polymetallic arsenic-bearing solids. During the high-temperature stage, arsenic is converted to volatile species such as As_2_O_3_ and As_4_O_6_ and captured before dilution. Wet processing then dissolves, oxidizes, and precipitates/crystallizes the captured material to separate arsenic from valuable metals [140,141]. Compared with conventional cooling and dust collection, high-temperature filtration may limit the dispersion of arsenic among the off-gas, melt, and final residues. Its industrial use, however, depends on filter durability and on the leaching and final stabilization of arsenic-rich dust. Major reactions are listed in Table 21, and the process flow is shown in Figure 20.

As shown in Table 21, high-temperature oxidation converts arsenic sulfides or elemental arsenic to volatile As_2_O_3_, which enters the off-gas as species such as As_4_O_6(g)_. Cooling or filtration capture then yields solid As_2_O_3_ enriched in high-temperature filter dust or condensate. In the wet stage, As_2_O_3_ can be hydrated to H_3_AsO_3_, oxidized to H_3_AsO_4_, and precipitated with Fe^3+^ as scorodite or ferric arsenate. Valuable-metal oxides such as CuO in filter dust can enter the solution by acid leaching, coupling arsenic separation/immobilization with valuable metal recovery [140,141].

This process captures arsenic-bearing particles at high temperature, treats the collected material hydrometallurgically, and stabilizes or disposes of the remaining arsenic. Arsenic-bearing melts, slag droplets, and off-gas particulates are treated under high-temperature oxidizing conditions, converting arsenic sulfides and related phases to volatile As_2_O_3_ while ceramic filters or high-temperature capture media enrich arsenic-bearing particles. Captured solids enter a wet reactor, where acid leaching converts As_2_O_3_ to As(III) solution and subsequent oxidation produces As(V). Cu, Fe, Zn, Pb, and Sb concentrations must be measured before selecting the metal-recovery step [142]. Arsenic-rich leachate can then be stabilized through lime neutralization, sulfide precipitation, ferric coprecipitation, or scorodite crystallization to form calcium arsenate, As_2_S_3_, ferric arsenate, or scorodite solids [143].

#### 3.3.5. Comparison of Integrated Pyrometallurgical–Hydrometallurgical Processes

Data are listed in Table 22.

**Table 22:** Comparison of integrated pyrometallurgical–hydrometallurgical processes.

Integrated Route	Representative Feedstock	Key Pretreatment/Thermal Conditions	Key Wet-Treatment Conditions	As Separation/Removal Performance	Valuable Metal Recovery/Enrichment	Final As Destination	Development Status
Acid roasting–water leaching	Gold concentrate	H_2_SO_4_-assisted roasting at 400 °C for 4 h	Water leaching at 70 °C for 60 min	81.43% As leaching; 92.03% overall As removal from solid [69]	Fe leaching 80.02%; representative maxima: Cu 70.10%, Zn 81.93%, Pb 50.48%; Au/Ag enriched in final residue [143]	Arsenic-bearing leachate	Laboratory-scale
O_2_-enriched roasting–H_2_SO_4_/NaCl leaching	Scrap copper anode slime	O_2_-enriched roasting; representative roasting time 60 min	H_2_SO_4_–NaCl selective leaching under optimized conditions	98.6% As separation in scale-up experiment	Cu 99.3%, Ni 96.9%, Pb 98.1%, Sn 98.9%; precious-metal enrichment >570%	As-containing leachate returned to electrolyte purification	Scale-up experiment
NaOH roasting–water leaching–synergistic acid leaching	Copper anode slime	NaOH/slime = 1.0; roasting 700 °C, 2 h	Water leaching followed by 2.00 M H_2_SO_4_ + 0.33 M tartaric acid + 0.44 M H_2_O_2_, S/L = 25 g L^−1^, 80 °C	95.20% As dissolved during water-leaching stage	Final removal: Cu 99.68%, Ni 81.99%, Sb 99.94%; retention: Ag 99.98%, Au 99.50%, Pd 91.00%	Arsenic-rich aqueous stream	Laboratory-scale
Leaching–purification–electrowinning	Arsenic-rich copper-smelting flue dust + copper-smelting waste acid	No separate smelting step in the cited study; water-leach pretreatment before electrolyte purification	Electrowinning: 200 A m^−2^, H_2_SO_4_ 120 g L^−1^, additive 120 g t^−1^ Cu, 50 °C, electrode spacing 45 mm, endpoint Cu^2+^ 30.50 g L^−1^	Overall As removal percentage NR in the cited abstract; As removed during leaching/purification	6N high-purity Cu; current efficiency 99.65%; cell voltage 1.87 V; energy consumption 1582.93 kWh t^−1^ Cu [139]	Arsenic-bearing purification stream/residue	Laboratory/engineering validation using industrial raw materials

### 3.4. Thermodynamic and Kinetic Basis of Arsenic Separation

Thermodynamic and kinetic data allow arsenic-separation processes to be compared quantitatively rather than only through reaction schemes. In pyrometallurgical systems, arsenic volatilization and condensation are governed by Gibbs free-energy changes, vapor-pressure differences, gas-phase equilibria, oxygen potential, and phase-partition relationships. In hydrometallurgical systems, acid–base equilibria, complex-formation constants, solubility products, redox potentials, and mineral dissolution thermodynamics determine arsenic speciation and precipitation selectivity [144]. However, thermodynamic favorability alone does not ensure rapid or selective separation because diffusion through product layers, interfacial chemical reactions, nucleation and crystal growth, mass transfer, and competitive precipitation may control the overall process rate. Table 23 compiles representative thermodynamic and kinetic parameters together with operating conditions and reported outcomes.

**Table 23:** Representative thermodynamic and kinetic evidence for arsenic separation processes.

Process	Feed/System	Thermodynamic/Kinetic Basis	Representative Quantitative Parameter	Process Outcome	Scale
Low-temperature roasting	Cu-smelting flue dust	Thermodynamic driving force for conversion of arsenate/As sulfides to As_2_O_3_ [144]	300–400 °C; 2–3 h; 5% coal; H_2_SO_4_ 0.2–0.3 mL g^−1^	~98% As volatilized; residue 0.57% As; As_2_O_3_ purity 99.05%	Lab
Vacuum distillation	Industrial yellow phosphorus	Saturated vapor pressure, separation coefficient, vapor–liquid equilibrium + AIMD [132]	As decreased from 136 to 1.09 mg kg^−1^	>99% As removal	Lab
Ultrasound-assisted alkaline leaching	Cu-smelting flue dust	Shrinking-core/chemical-reaction-controlled kinetics; Arrhenius analysis	k = 0.0017, 0.0032, 0.0071 min^−1^ at 40, 60, 80 °C; Ea = 36.43 kJ mol^−1^	~99% As leaching	Lab
Sulfide precipitation	Cu–As electrolyte solution	Ksp, complexation equilibrium and competitive H_2_S consumption	Cd^2+^/Zn^2+^ decreased As(V) precipitation by 2.75/1.47%; Bi^3+^/Sn^2+^ increased it by 2.58/8.14% [138]	Reaction-time control reduces As coprecipitation with Cu	Lab + real solution validation

### 3.5. Overall Comparison of Arsenic Separation Technologies

Data are listed in Table 24.

**Table 24:** Overall comparison of arsenic separation technologies.

Category	Preferred Feedstock	Representative As Performance	Energy Demand	Reagent Demand	Main As Destination	Main Secondary Stream	Key Safety/Environmental Issue	Overall Maturity/Application
Pyrometallurgical separation	High-As solids, sulfide concentrates, dusts, alloys	High-throughput separation; representative values summarized in Table 5	High	Low–moderate	As_2_O_3_-rich dust/condensate/arsenic-bearing slag	Off-gas, dust, calcine/slag	As_2_O_3_ dust, SO_2_, high-T operation, As recirculation	Established for selected conventional roasting/smelting systems; process-specific evidence required [144]
Hydrometallurgical separation	Waste acids, leachates, wastewater, soluble dusts	High As removal/selectivity possible; see Table 16	Low–moderate	Moderate–high	As_2_S_3_, Fe–As sludge, scorodite, loaded media	Wastewater, sludge, regenerant	Acid/alkali consumption, H_2_S, sludge stability	Established for several wastewater-treatment routes; advanced routes remain feed-specific
Integrated pyrometallurgical–hydrometallurgical route	Complex polymetallic secondary materials	Combines As control with metal recovery; see Table 21	Moderate–high	Moderate–high	As condensate + stabilized residue + recovered metal	Off-gas + wastewater + solid residue	Multistage secondary pollution and process-control complexity	Promising for complex feeds; industrial evidence varies strongly by flowsheet
Deep purification/product-oriented recovery	Arsenic-rich intermediate, crude As_2_O_3_, crude metallic As	Upgrading toward refined As_2_O_3_/high-purity As	Moderate–high	Process-dependent	Refined As_2_O_3_/metallic As/≥5N As	Distillation residue/purification waste	Trace impurity control, volatile As compounds	Suitable where product value offsets purification cost

### 3.6. Environmental Stability of Arsenic-Bearing Solid Residues

Arsenic precipitation and immobilization should be judged by both initial removal efficiency and the long-term stability of the resulting solid. Calcium arsenates, ferric arsenates, As_2_S_3_, Fe–As sludge, and scorodite differ substantially in crystallinity, solubility, aging behavior, and sensitivity to pH and redox conditions. Calcium arsenate phases may undergo carbonation and phase transformation during aging; amorphous or poorly crystalline Fe–As precipitates may release arsenic as Fe-bearing phases recrystallize; and As_2_S_3_ is vulnerable to enhanced dissolution and oxidation under alkaline and oxic conditions. Well-crystallized scorodite generally provides comparatively favorable arsenic retention under mildly acidic and oxidizing conditions, but its stability decreases when environmental pH and redox conditions move outside this range [145]. No arsenic-bearing residue can be considered universally stable; its formation conditions, mineralogy, leaching behavior, aging history, and expected disposal environment must be specified [146,147]. Detailed information is shown in the Table 25.

**Table 25:** Comparative environmental stability of representative arsenic-bearing solid residues.

Solid As Phase	Solubility/Leachability	Aging Behavior	Acidic Conditions	Alkaline Conditions	Oxic Conditions	Reducing/Anoxic Conditions	Overall
Calcium arsenate	Condition-dependent; long-term leaching remains a concern [145]	Carbonation and phase transformation may remobilize As	Acid dissolution can increase As release	Stability strongly depends on pH, Ca/As and CO_2_ exposure	Not intrinsically stable	Requires further investigation	Not preferred as a universally stable terminal residue
Ferric arsenate	Generally lower solubility in appropriate acidic range; strongly structure-dependent	Amorphous phases may recrystallize and alter As retention	Relatively favorable under moderately acidic conditions	Stability generally decreases outside favorable pH range	Condition-dependent	Redox stability should be considered	More stable than Ca–As in many systems, but strongly phase-dependent
As_2_S_3_	Relatively low solubility under suitable acidic/low-O_2_ conditions	Can oxidize or dissolve with environmental changes	Generally more favorable than alkaline conditions	Dissolution increases substantially	Oxidation enhances As release	Comparatively more persistent but pH-dependent	Better treated as an intermediate unless disposal conditions are controlled
Fe–As sludge	Highly variable because of heterogeneous/amorphous composition	Ferrihydrite/Fe-phase aging can alter As retention	Often more stable in acidic than neutral conditions	Increased remobilization risk	Depends on Fe mineralogy	Requires redox	Long-term stability cannot be inferred from initial removal efficiency
Scorodite, FeAsO_4_·2H_2_O	Comparatively low for well-crystallized material under suitable conditions	Crystallinity and secondary transformation affect stability	Favorable mainly under mildly acidic conditions	As release increases	Most favorable under mildly acidic oxic conditions	Stability decreases under reducing conditions	One of the more favorable As phases, but only under defined synthesis and exposure conditions [102]

### 3.7. Engineering, Environmental, and Economic Considerations

Process selection must account for arsenic-removal efficiency, energy and reagent consumption, secondary waste generation, process safety, and economic feasibility. Pyrometallurgical processes are generally characterized by high treatment capacity and compatibility with arsenic-rich solid feeds, but elevated temperatures increase energy demand and require stringent capture of As_2_O_3_-bearing dust, volatile arsenic species, and SO_2_-containing off-gas. Hydrometallurgical processes operate under comparatively mild thermal conditions but may require substantial consumption of acids, alkalis, oxidants, ferric salts, or sulfide reagents and can generate arsenic-bearing wastewater and sludge that require further treatment.

Combined pyrometallurgical–hydrometallurgical flowsheets can separate arsenic and recover valuable metals, but their longer process chains and multiple solid–liquid and gas-cleaning operations increase capital and operating complexity. Processes involving H_2_S, AsH_3_, volatile As_2_O_3_, strong acids or alkalis, or pressurized or vacuum equipment require additional safety controls. A credible industrial assessment requires feed-specific mass and energy balances, reagent demand, wastewater and off-gas generation, residue stability, valuable-metal recovery, product value, and capital and operating costs; arsenic-removal efficiency alone is insufficient.

Direct numerical comparison among different studies remains subject to uncertainty because the arsenic concentration, mineralogical occurrence, particle size, reagent basis, reactor configuration, analytical method, and endpoint definition differ among feedstocks and laboratories. The values compiled here are source-specific and should not be treated as universal optima. Future studies should report feed mineralogy, operating conditions, mass balance, uncertainty, and process scale in a consistent form to permit cross-study comparison. Detailed information is shown in the Table 26.

**Table 26:** Engineering and environmental considerations for representative arsenic treatment routes.

Route	Energy Demand	Main Reagents	Wastewater	Off-Gas	Solid Residue	Main Safety Issue	Economic Consideration	Development Status
Roasting	High	O_2_/air, additives	Low–moderate	As_2_O_3_/SO_2_-bearing	Calcine/dust	Toxic dust/off-gas	Energy + gas cleaning [146,147]	Case-based
Vacuum distillation	Moderate–high	Limited reagent	Low	As vapor/condensate	Refined residue	Vacuum/high T/As vapor	Equipment cost	Case-based
Acid leaching	Moderate	Acid + oxidant	High	Usually limited	Leach residue	Corrosive liquor	Acid + wastewater	Case-based
Sulfide precipitation	Low thermal	Na_2_S/H_2_S	Moderate	H_2_S risk	As_2_S_3_	H_2_S toxicity	Sulfide + gas control	Case-based
Ferric precipitation	Low thermal	Fe salt + lime	Moderate–high	Low	Fe–As sludge	Sludge handling	Reagent/sludge disposal	Case-based
Integrated route	Moderate–high	Multiple	Moderate–high	Process-dependent	Multiple residues	Multistage hazards	CAPEX/OPEX complexity	Case-based

## 4. Preparation Technologies for Arsenic Products

Arsenic has distinctive chemical reactivity and semiconductor properties. Historically, it was used mainly in conventional applications such as wood preservatives, pesticides, pigments, and alloying additives. However, because inorganic arsenic compounds have substantial toxicity and potential for environmental accumulation, some conventional arsenic-containing products are now tightly restricted and are gradually being replaced by lower-toxicity alternatives. Current development concentrates on high-purity and functional arsenic products rather than conventional low-value applications [148]. In this review, arsenic-containing products are selected according to their positions in the recovery–purification–utilization chain of arsenic-bearing metallurgical and secondary resources rather than simply by their commercial uses. Crude and refined As_2_O_3_ and metallic arsenic are considered primary recovery products or intermediate arsenic carriers; high-purity arsenic and AsH_3_ represent deeply purified arsenic sources for advanced applications; and GaAs, InAs, and Zn_3_As_2_ are discussed as representative downstream high-value arsenide materials that can utilize purified arsenic derived from secondary resources. Cu–As and Pb–As alloys are included mainly because they can serve as metallurgical arsenic enrichment or intermediate phases during arsenic separation and transfer, rather than as priority end products for expanded arsenic consumption. The products are organized here by their position in the sequence from arsenic recovery and enrichment to purification and controlled end use.

### 4.1. Preparation of Arsenic Trioxide

Arsenic trioxide (As_2_O_3_) is the principal recovered arsenic product obtained during the separation and processing of arsenic-bearing resources. It can be recovered from metallurgical flue dust and arsenic-rich intermediate materials and can also serve as an intermediate feedstock for metallic arsenic, high-purity arsenic, and selected arsenic compounds [121]. Crude and refined As_2_O_3_ differ in purity, downstream use, impurity specifications, and safety requirements.

#### 4.1.1. Crude Arsenic Trioxide

Crude As_2_O_3_ is obtained principally from oxidative roasting or smelting-volatilization of arsenic-bearing sulfide ores, arsenic-bearing metallurgical flue dust, arsenic-bearing copper-smelting flue dust, and arsenic-rich intermediate materials. The basic principle is to convert arsenic sulfides, elemental arsenic, or low-valence arsenic-bearing species in the feed to readily volatile As_2_O_3_ under oxidizing conditions. The vapor then migrates with the off-gas and is collected as crude As_2_O_3_ by cooling, condensation, and dust collection [149,150]. Because this process aims mainly for volatilization-based arsenic enrichment, the crude product typically contains about 90–98% As_2_O_3_ by mass and still contains impurity elements such as S, Sb, Bi, Pb, Fe, and Cu. This approach has a simple flowsheet, high treatment capacity, and adaptability to complex arsenic-bearing materials, making it suitable for rapid enrichment of arsenic in smelting by-product streams. Its relatively high impurity content, however, generally precludes direct use in high-purity arsenic or semiconductor-material applications, and further refining is required.

#### 4.1.2. Refined Arsenic Trioxide

**Figure 21:**
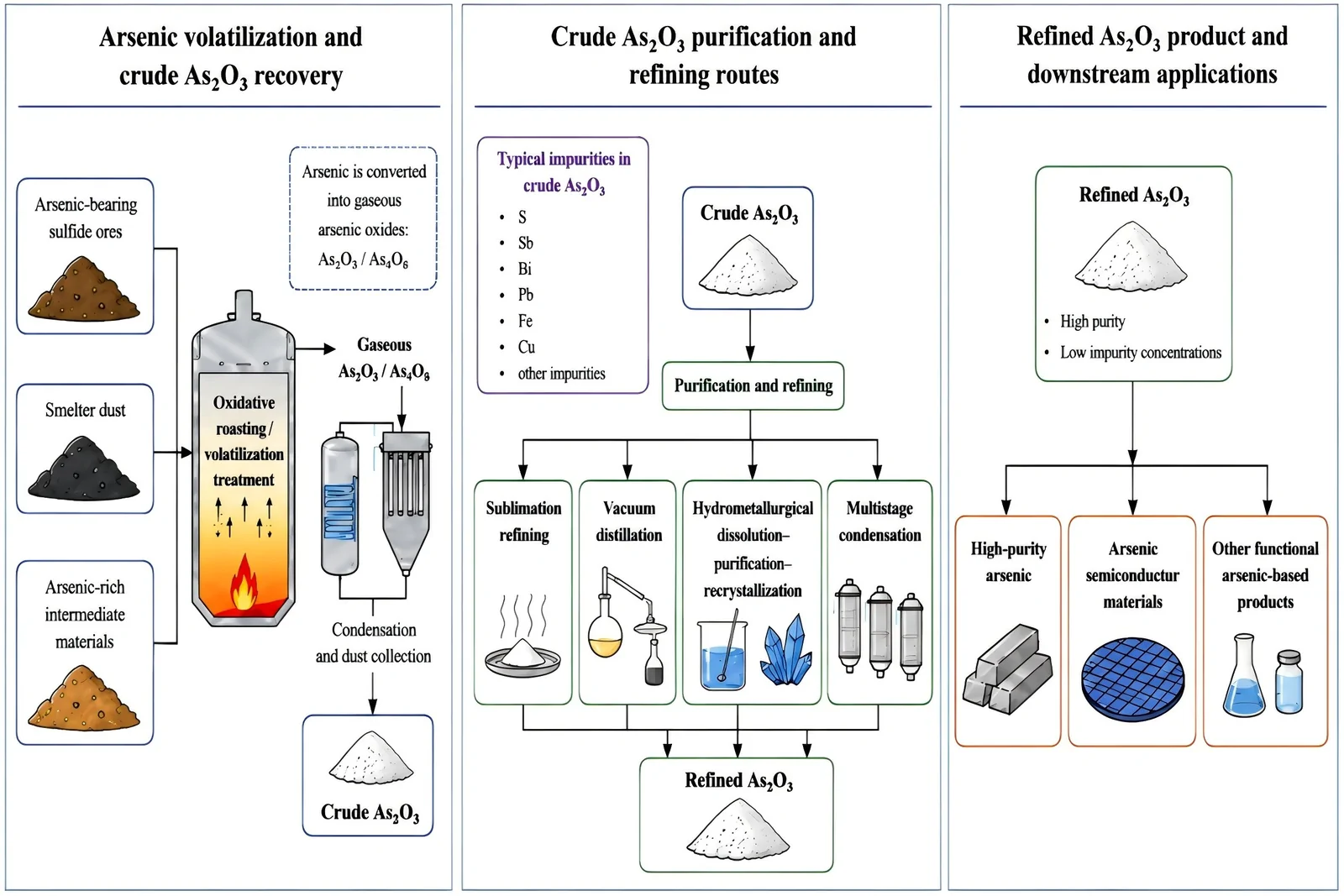
Simplified production and purification routes for crude and refined arsenic trioxide. AI was used for this figure, specifically GPT 5.5, mainly for logical sorting.

Refined As_2_O_3_ is generally produced from crude As_2_O_3_ or arsenic-rich flue dust through a combination of sublimation refining, vacuum distillation, hydrometallurgical purification, recrystallization, and multistage condensation. Crude As_2_O_3_ from metallurgical arsenic-bearing materials typically contains S, Sb, Bi, Pb, Fe, Cu, and other impurities and is unsuitable for direct use as a high-purity product. During sublimation or vacuum treatment, Fe- and Cu-bearing impurities remain preferentially in the residue because they are less volatile than As_2_O_3_; staged condensation separates arsenic oxide from co-volatilized impurity-bearing fractions. Some Sb, Bi, and Pb compounds co-volatilize with arsenic, so their removal may require repeated sublimation, fractional condensation, or complementary hydrometallurgical purification. Wet purification removes additional Fe, Cu, Pb, Sb, and other metallic impurities by dissolution followed by selective precipitation, redox adjustment, solvent extraction, ion exchange, or recrystallization. Sulfur-bearing species are controlled through oxidation, volatilization, precipitation, and repeated refining, depending on their chemical form. Reported multistage processes upgrade crude As_2_O_3_ from approximately 90–98 wt% to refined As_2_O_3_ at approximately 99–99.999% (2N–5N). The refined product is a more suitable feedstock for metallic arsenic and subsequent high-purity arsenic production. Figure 21 summarizes the production and purification methods for crude and refined arsenic trioxide.

Figure 21 summarizes the principal pathway from crude As_2_O_3_ recovery to refined product. First, arsenic-bearing sulfide ores, metallurgical flue dust, and arsenic-rich intermediate materials are subjected to oxidative roasting or volatilization treatment, converting arsenic to gaseous As_2_O_3_/As_4_O_6_; crude As_2_O_3_ is then collected by condensation and dust collection [151]. Because the crude product commonly contains S, Sb, Bi, Pb, Fe, Cu, and other impurities, it is further purified by sublimation refining, vacuum distillation, hydrometallurgical dissolution–purification–recrystallization, and multistage condensation. Selective volatilization, impurity separation, and recrystallization yield refined As_2_O_3_ with low impurity concentrations. The refined oxide can then be used to prepare high-purity arsenic, arsenide semiconductors, and other functional arsenic products.

### 4.2. Metallic Arsenic and High-Purity Arsenic

High-purity arsenic generally refers to elemental arsenic with a purity of ≥5N and is used as the arsenic source for Group III–V compound semiconductors such as GaAs, InAs, and InGaAs. Its production from metallurgical arsenic-bearing resources is not a single-step conversion but a multistage separation and purification process [152]. Arsenic-bearing flue dusts, sulfide residues, and other secondary materials are first subjected to roasting, volatilization, condensation, or wet separation to obtain arsenic-rich intermediates, typically crude As_2_O_3_. After further sublimation, vacuum distillation, hydrometallurgical purification, and recrystallization, refined As_2_O_3_ can be obtained and subsequently reduced to metallic arsenic. The metallic arsenic is then subjected to deeper purification by chlorination–distillation–reduction, repeated sublimation or vacuum distillation, zone refining, or vapor-phase precursor purification to produce high-purity arsenic.

**Figure 22:**
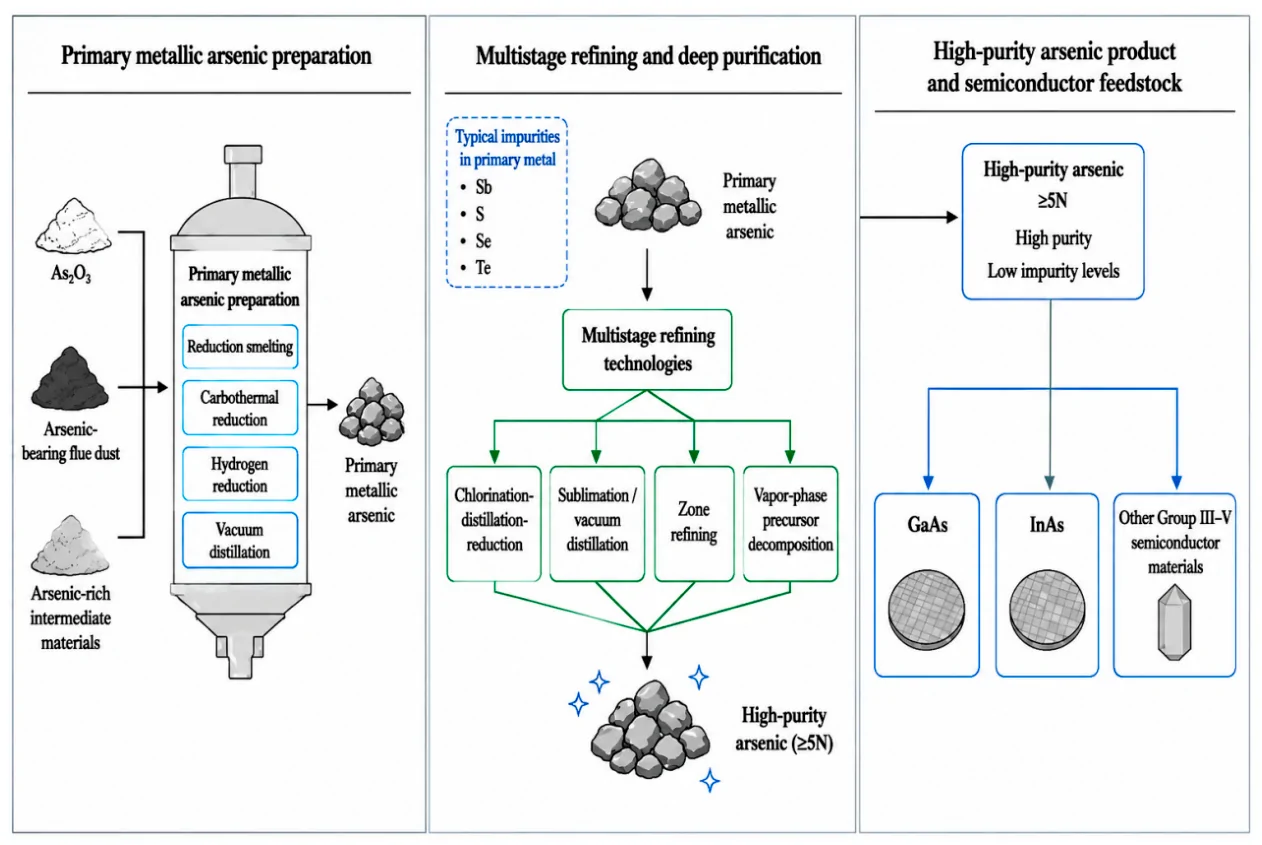
Simplified route for preparing metallic arsenic and converting it to high-purity arsenic. AI was used for this figure, specifically GPT 5.5, mainly for logical sorting.

The principal challenge in this purification chain is the progressive removal of trace Sb, Bi, Pb, Se, Te, S, Fe, and Cu. Fe and Cu are comparatively nonvolatile and can be preferentially retained in residues during sublimation or vacuum treatment and can also be removed through hydrometallurgical purification. Sb, Bi, and Pb are more difficult to separate because their volatile compounds may partially accompany arsenic during thermal purification, requiring fractional condensation, repeated distillation, or complementary chemical separation. S, Se, and Te require deeper purification because of their chemical similarity and potential influence on semiconductor-grade arsenic quality; repeated vacuum/sublimation refining, chlorination–distillation–reduction, and zone refining are therefore employed in the final purification stages. A typical sequence proceeds from crude As_2_O_3_ (approximately 90–98%) to refined As_2_O_3_ (approximately 99–99.999%), metallic arsenic, and finally high-purity arsenic of ≥5N. For semiconductor applications, acceptance depends on individual trace-impurity concentrations as well as total arsenic purity. Figure 22 illustrates the preparation of metallic arsenic and its conversion to high-purity arsenic.

As summarized in Figure 22, the conversion of secondary arsenic-bearing resources into semiconductor-grade arsenic involves successive enrichment, intermediate purification, reduction, and deep refining rather than direct conversion. Each stage raises arsenic purity, from crude and refined As_2_O_3_ through metallic arsenic to ≥5N material. Impurity removal correspondingly changes from bulk Fe, Cu, Pb, and S to trace Sb, Bi, Se, Te, and other semiconductor-sensitive elements. Detailed information is shown in the Table 27.

**Table 27:** Representative purification chain from metallurgical arsenic-bearing materials to high-purity arsenic.

Stage	Feed/Intermediate	Typical Purity	Major Impurities	Main Purification Step	Product
Enrichment	Arsenic-bearing dust/residue	Feed-dependent	Cu, Fe, Pb, Zn, Sb, Bi, S	Roasting/leaching/volatilization [153]	Arsenic-rich intermediate
Primary recovery	Crude As_2_O_3_	~90–98%	S, Sb, Bi, Pb, Fe, Cu	Condensation + primary purification	Crude As_2_O_3_
Refining	Refined As_2_O_3_	~99–99.999%	Trace metals/S-bearing species	Sublimation/vacuum/hydrometallurgical purification	Refined As_2_O_3_
Reduction	Metallic As	>90% after direct recovery; up to 99.99% (4N) after further vacuum refining	Sb, Bi, Pb, Se, Te, S	Reduction + vacuum refining	Metallic As
Deep purification	High-purity As	≥5N [154]	Trace Sb, Bi, Se, Te, Pb, S	Zone refining/vacuum/chlorination route	Semiconductor-grade As

### 4.3. Arsenic-Containing Alloys

#### 4.3.1. Copper–Arsenic Alloys

**Figure 23:**
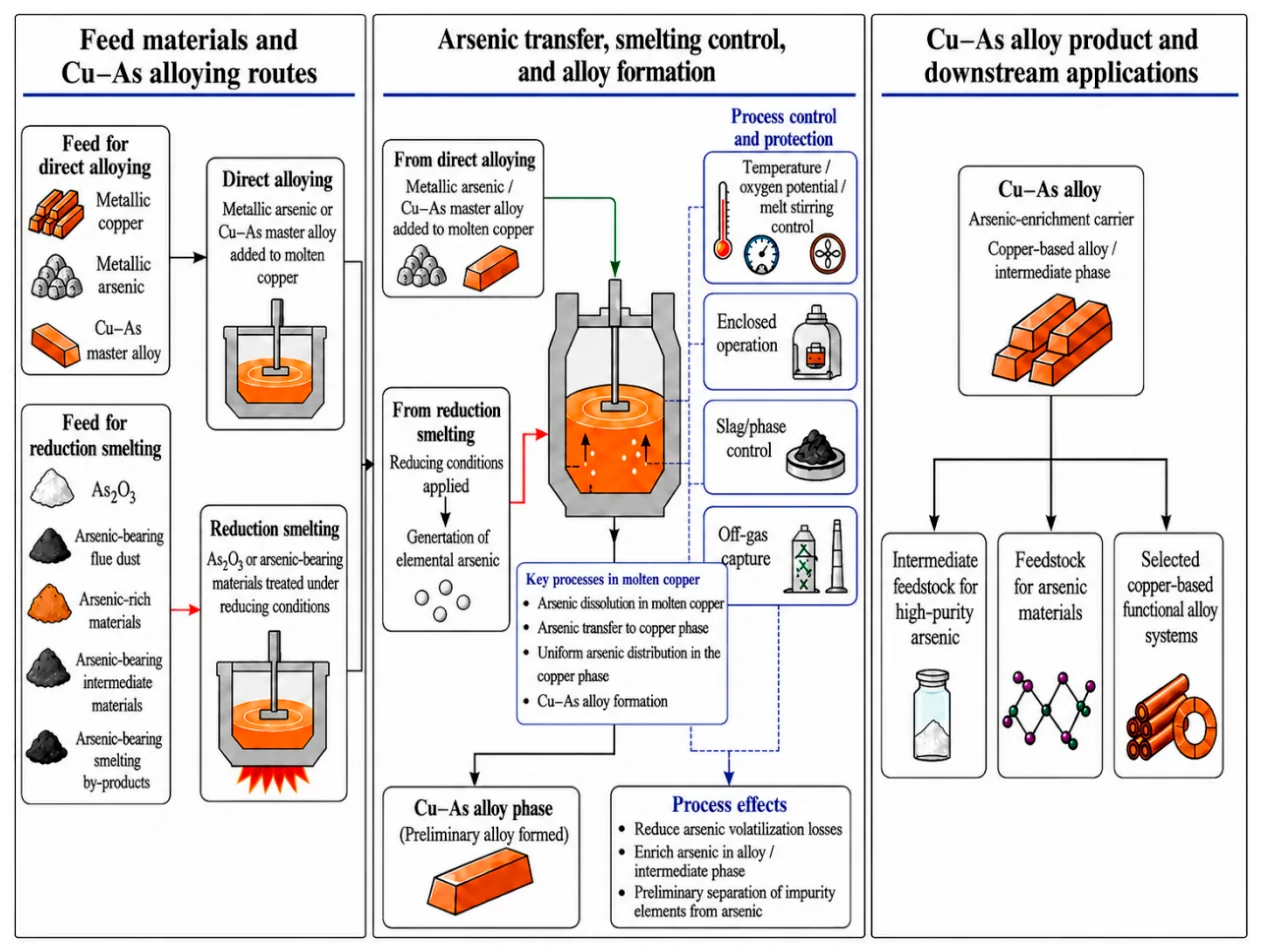
Schematic of copper–arsenic alloy preparation. AI was used for this figure, specifically GPT 5.5, mainly for logical sorting.

Copper–arsenic (Cu–As) alloys are commonly prepared from metallic copper, arsenic-bearing intermediate materials, or arsenic-bearing smelting by-products by direct alloying or reduction smelting [153,155]. In direct alloying, metallic arsenic or a Cu–As master alloy is added to molten copper; the temperature, oxygen potential, and stirring rate are set so that arsenic dissolves and distributes uniformly in the copper phase. In reduction smelting, As_2_O_3_, arsenic-bearing flue dust, or arsenic-rich materials are treated under reducing conditions to generate elemental arsenic, which subsequently migrates into the molten copper to form a Cu–As alloy phase. Enclosed smelting, slag control, and off-gas capture limit arsenic volatilization, concentrate arsenic in the copper alloy or intermediate, and remove part of the impurity load. The resulting Cu–As alloy can serve as an arsenic enrichment carrier and intermediate feedstock for high-purity arsenic and arsenide materials, and it can be used in selected copper-based functional alloy systems. Figure 23 shows a schematic of Cu–As alloy preparation.

#### 4.3.2. Lead–Arsenic Alloys as Arsenic Enrichment Intermediates

**Figure 24:**
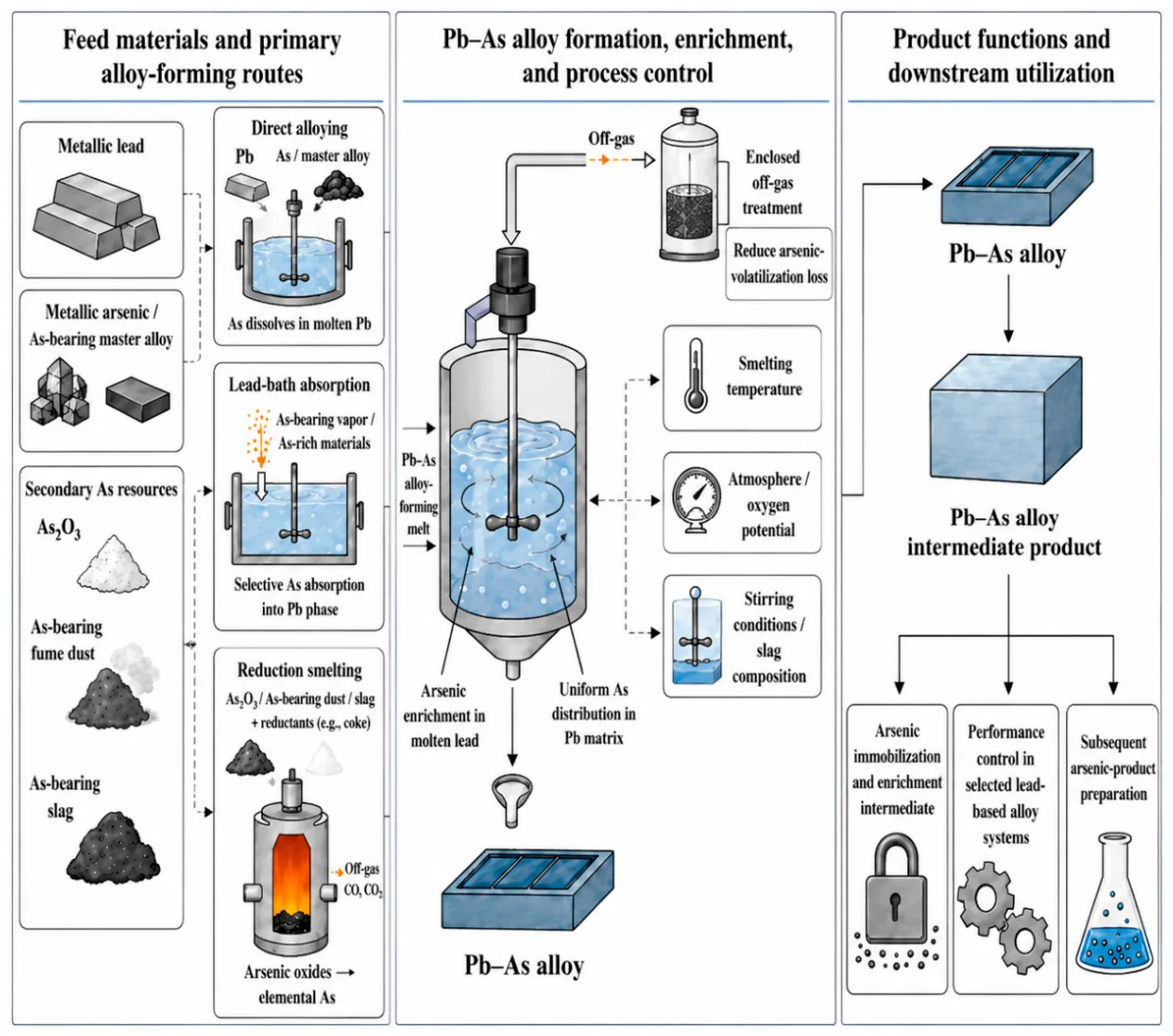
Schematic of lead–arsenic alloy preparation. AI was used for this figure, specifically GPT 5.5, mainly for logical sorting purposes.

Lead–arsenic (Pb–As) alloys are generally prepared from metallic lead, metallic arsenic, or arsenic-bearing metallurgical intermediates by direct alloying, lead-bath absorption, or reduction smelting. In direct alloying, metallic arsenic or an arsenic-bearing master alloy is added to molten lead, and the smelting temperature, atmosphere, and stirring conditions are controlled to dissolve arsenic fully and distribute it uniformly in the lead matrix. Lead-bath absorption exploits the selective capacity of molten lead to absorb arsenic, transferring arsenic from arsenic-bearing vapor or arsenic-rich materials into the lead phase to form a Pb–As alloy. Secondary resources such as As_2_O_3_, arsenic-bearing flue dust, and arsenic-bearing slag can also be treated by reduction smelting to convert arsenic oxides to elemental arsenic and enrich the arsenic in molten lead. During preparation, the oxygen potential, slag composition, and enclosed off-gas treatment are adjusted to limit volatilization and increase arsenic recovery into the alloy. For secondary-resource treatment, Pb–As alloys function mainly as arsenic-enrichment intermediates, not as preferred end products. Their inclusion in this review is intended to illustrate the transfer and concentration of arsenic into a controllable metallic phase during pyrometallurgical processing. Further separation or refining is generally required if the contained arsenic is to enter higher-value product chains [156,157]. Because both Pb and As are toxic and conventional Pb–As applications are increasingly restricted, expanding Pb–As alloy production is not recommended. Figure 24 shows a schematic of Pb–As alloy preparation.

### 4.4. Arsenic Compounds

#### 4.4.1. Zinc Arsenide

**Figure 25:**
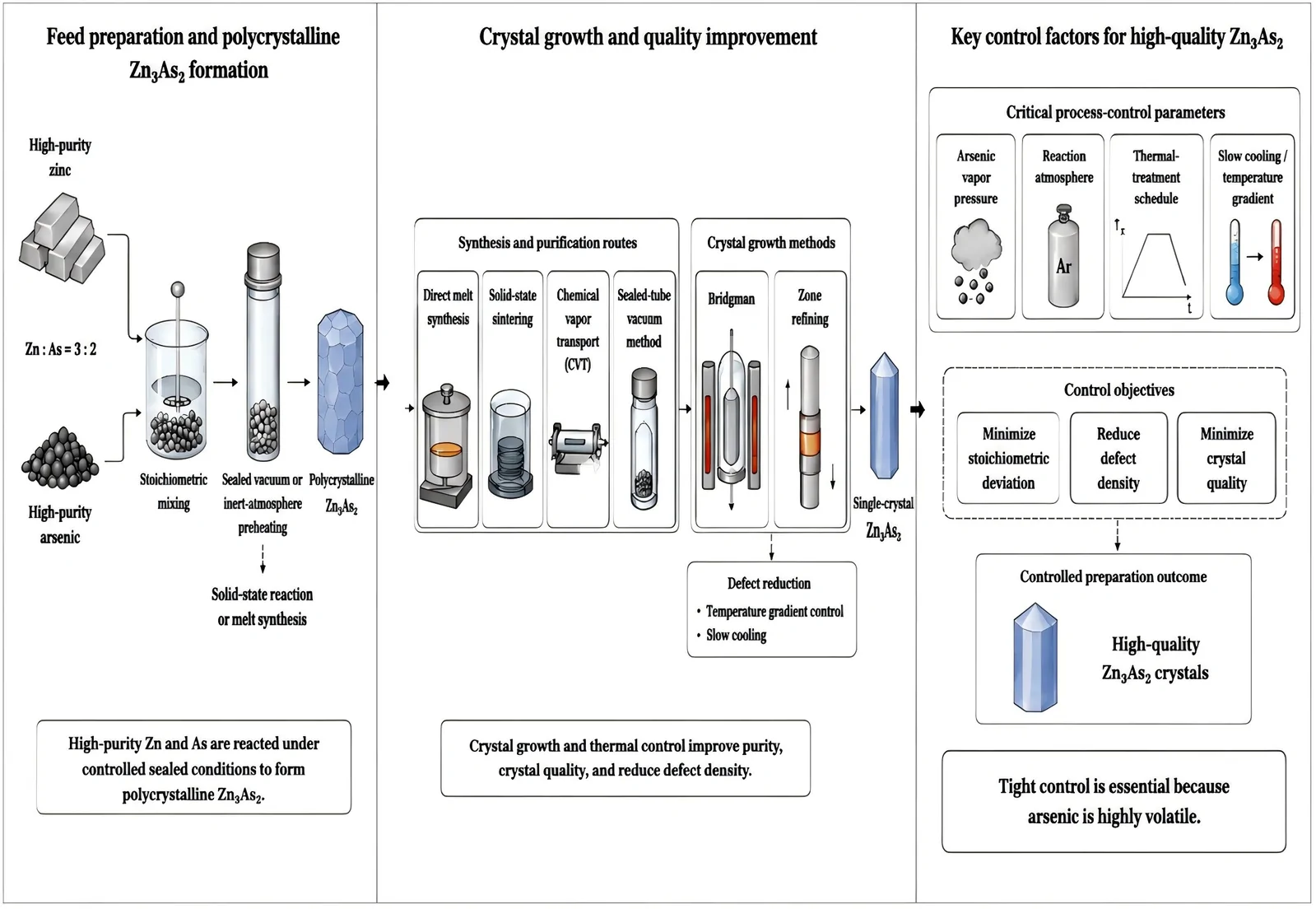
Schematic of zinc arsenide preparation. AI was used for this figure, specifically GPT 5.5, mainly for logical sorting.

Zinc arsenide (Zn_3_As_2_) is a typical Group II–V compound semiconductor [158,159]. It is commonly prepared from high-purity zinc and high-purity arsenic by solid-state reaction or melt synthesis. The reactants are first mixed at the stoichiometric ratio (Zn:As = 3:2) and heated in a sealed vacuum or inert-atmosphere system to form polycrystalline Zn_3_As_2_. Besides direct melt synthesis, solid-state sintering, chemical vapor transport (CVT), and sealed-tube vacuum methods can produce material with higher purity and better crystal quality. Crystal growth commonly uses techniques such as the Bridgman method and zone refining; temperature-gradient control and slow cooling are used to reduce defect density. Because arsenic is highly volatile, arsenic vapor pressure, the reaction atmosphere, and the thermal-treatment schedule must be tightly controlled to minimize stoichiometric deviation and obtain high-quality Zn_3_As_2_ crystals. Figure 25 shows a schematic of Zn_3_As_2_ preparation.

#### 4.4.2. Arsine

**Figure 26:**
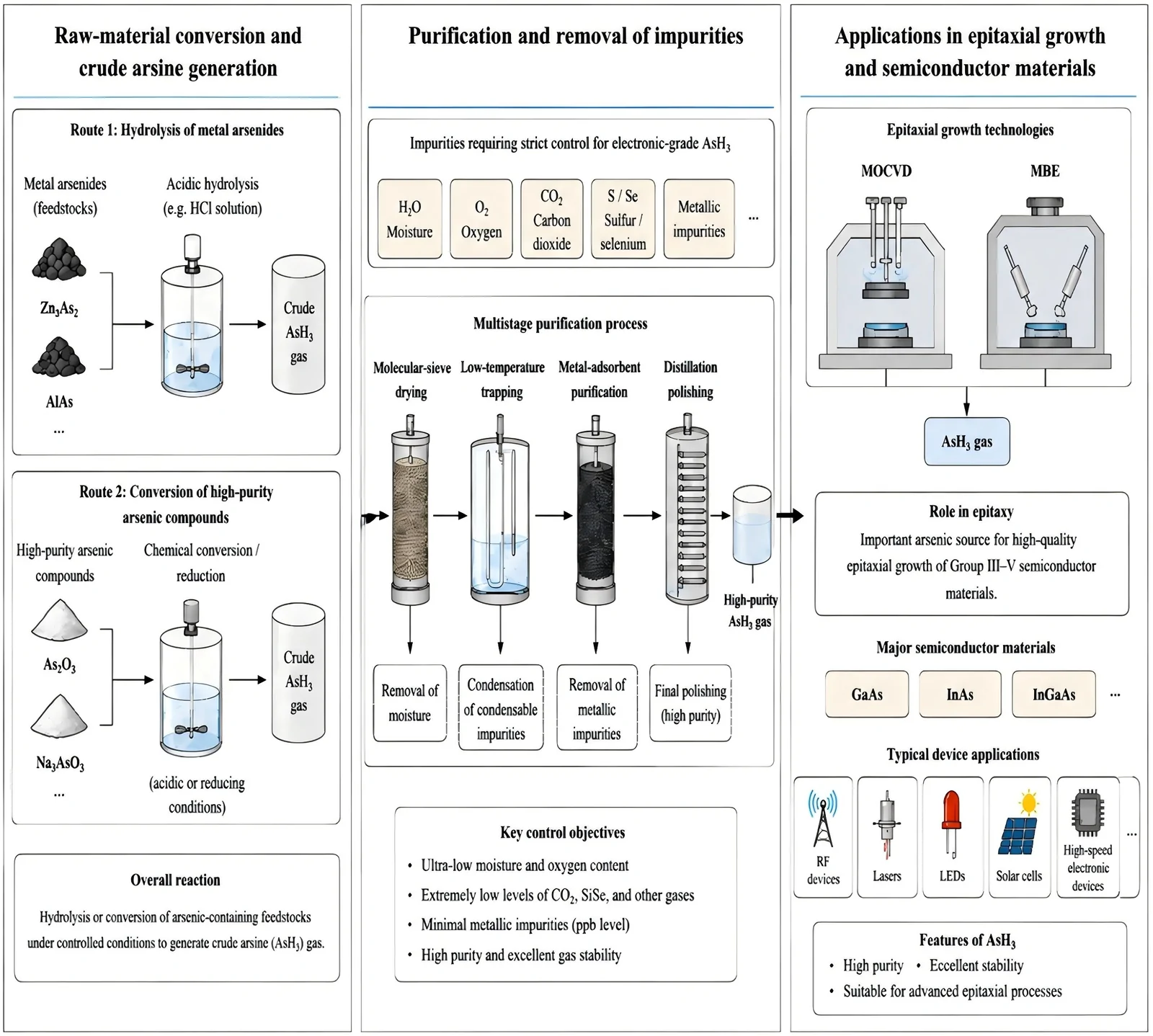
Schematic of arsine preparation. AI was used for this figure, specifically GPT 5.5, mainly for logical sorting.

Arsine (AsH_3_) is used as a vapor-phase precursor for epitaxial growth of high-purity arsenide semiconductors. Its preparation principally follows two routes: hydrolysis of metal arsenides and conversion of high-purity arsenic compounds. Traditional methods generally use metal arsenides such as Zn_3_As_2_ or AlAs as feedstocks, generating crude AsH_3_ through hydrolysis under acidic conditions. The gas is then purified by low-temperature condensation, drying, adsorption purification, and removal of metallic impurities. For electronic-grade AsH_3_, moisture, oxygen, CO_2_, sulfur and selenium species, and metallic impurities must be controlled. Electronic-grade AsH_3_ is therefore purified by molecular-sieve drying, low-temperature trapping, metal-adsorbent treatment, and distillation. High-purity AsH_3_ is used as the arsenic source in epitaxial processes such as metal–organic chemical vapor deposition (MOCVD) and molecular beam epitaxy (MBE) for Group III–V materials including GaAs, InAs, and InGaAs [160]. Figure 26 shows a schematic of arsine preparation.

GaAs and InAs are included only as downstream products of multistage arsenic recovery and purification; metallurgical arsenic-bearing residues cannot be used directly for semiconductor production. Arsenic recovered from metallurgical flue dusts, residues, or other secondary resources must first be separated and enriched, typically as crude As_2_O_3_ or an arsenic-rich intermediate, followed by refining to refined As_2_O_3_, metallic arsenic, high-purity elemental arsenic, or electronic-grade AsH_3_. Only after stringent removal of impurities such as Sb, Bi, Pb, Se, Te, S, Fe, and Cu can the purified arsenic source be considered suitable for III–V semiconductor production. They represent downstream uses of deeply purified secondary arsenic, not direct products of metallurgical arsenic-bearing wastes.

#### 4.4.3. Gallium Arsenide

**Figure 27:**
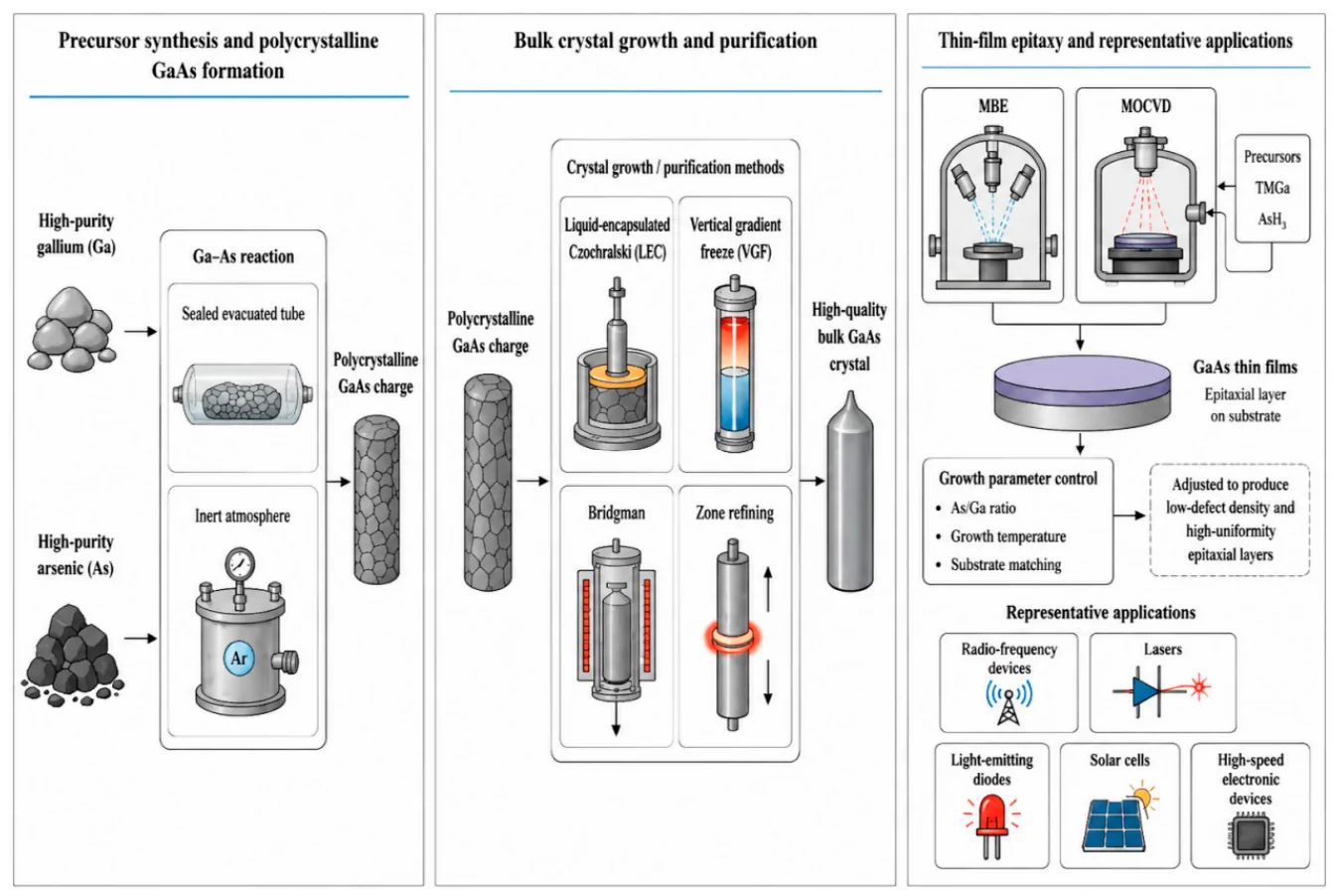
Schematic of gallium arsenide preparation. AI was used for this figure, specifically GPT 5.5, mainly for logical sorting.

Gallium arsenide (GaAs) is among the most widely used Group III–V compound semiconductors. It is commonly synthesized directly from high-purity gallium and high-purity arsenic and is subsequently processed by crystal-growth and epitaxial technologies to obtain high-quality materials. Bulk GaAs is generally produced by direct reaction of Ga and As in sealed evacuated tubes or under an inert atmosphere to form a polycrystalline charge, followed by crystal growth and purification by liquid-encapsulated Czochralski (LEC), vertical gradient freeze (VGF), Bridgman, or zone-refining methods [161]. Device-grade thin films are produced mainly by MBE and MOCVD [162]. Adjustment of the As/Ga ratio, growth temperature, and substrate matching is used to produce epitaxial layers with low defect density and high uniformity. Owing to its high electron mobility, direct bandgap, and excellent optoelectronic-conversion properties, GaAs is used primarily in radio-frequency devices, lasers, light-emitting diodes, solar cells, and high-speed electronic devices [13]. Figure 27 shows a schematic of GaAs preparation.

#### 4.4.4. Indium Arsenide

**Figure 28:**
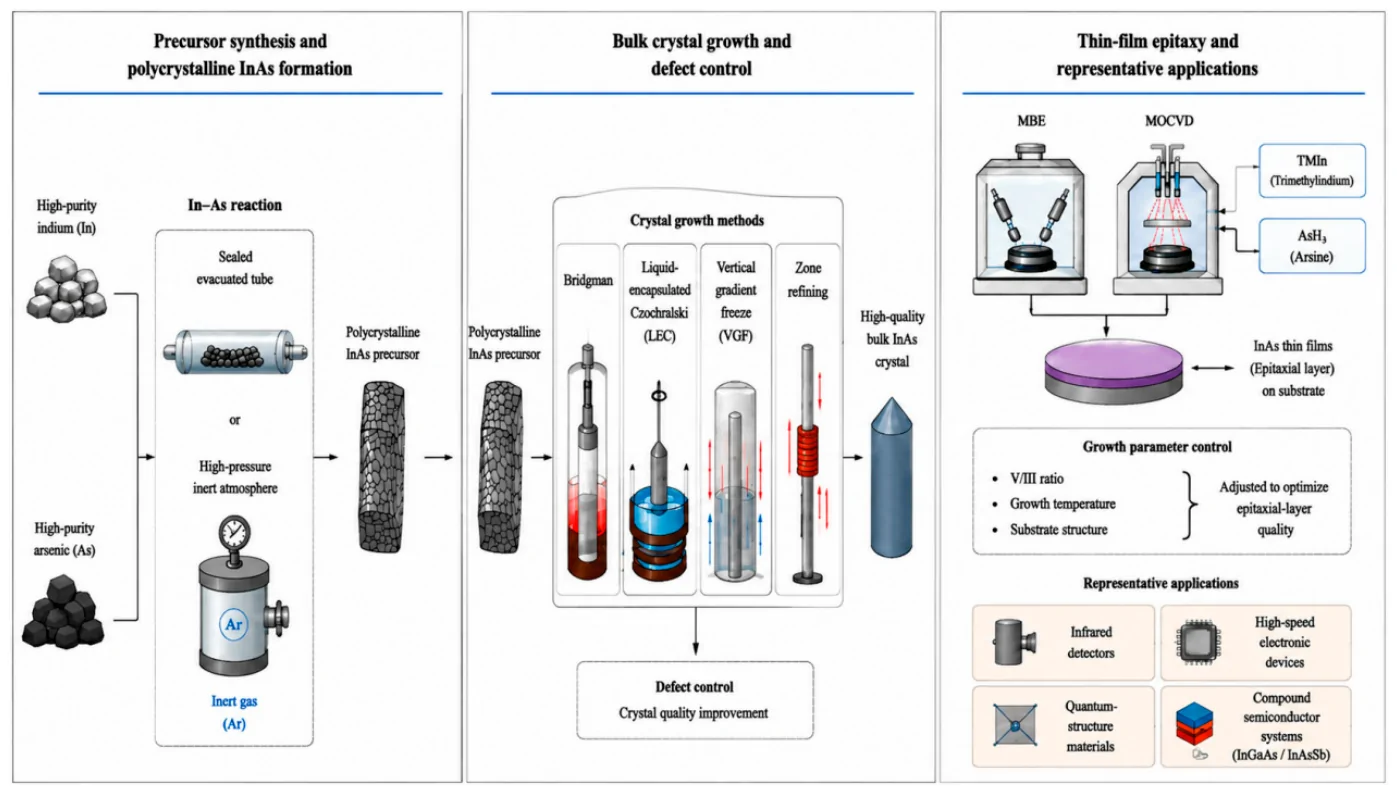
Schematic of indium arsenide preparation. AI was used for this figure, specifically GPT 5.5, mainly for logical sorting.

Indium arsenide (InAs) is a downstream arsenide product prepared from deeply purified arsenic. Its connection to secondary arsenic resources is indirect: separation, refining, and trace-impurity removal must first produce semiconductor-grade arsenic or AsH_3_. InAs is subsequently synthesized from high-purity indium and high-purity arsenic, followed by controlled crystal growth to obtain high-quality bulk materials. Bridgman, liquid-encapsulated Czochralski, vertical gradient freeze, or zone-refining methods are then used for crystal growth and defect control. InAs thin films are prepared mainly by MBE and MOCVD; trimethylindium (TMIn) and AsH_3_ are commonly used as precursors, and the V/III ratio, growth temperature, and substrate structure are adjusted to optimize epitaxial-layer quality. Owing to its narrow bandgap, high electron mobility, and excellent infrared response, InAs is used mainly in infrared detectors, high-speed electronic devices, quantum-structure materials, and compound semiconductor systems such as InGaAs and InAsSb [163,164]. Figure 28 shows a schematic of InAs preparation.

## 5. Conclusions and Outlook

For arsenic-rich sulfide concentrates, flue dusts, and other solid metallurgical residues, roasting- and smelting-based processes remain suitable for high-content arsenic separation and enrichment, provided that volatile arsenic species are effectively captured and the resulting dusts or slags are subsequently treated. For arsenic-bearing waste acids and leachates, ferric coprecipitation, sulfide precipitation, and scorodite crystallization provide more controllable routes for arsenic removal and stabilization, whereas adsorption and ion exchange are better suited to low-concentration treatment. Complex polymetallic secondary materials generally require integrated pyrometallurgical–hydrometallurgical flowsheets to combine arsenic control with recovery of Cu and other valuable metals.

Conventional roasting, ferric coprecipitation, and several wastewater-treatment methods are established at engineering scale. Advanced scorodite processing, selective combined flowsheets, high-temperature filtration, and direct production of high-purity arsenic from secondary materials still require more rigorous industrial validation. Future studies should match processes to feed mineralogy, quantify energy and reagent use, and verify long-term residue stability.

In the future, more basic research and applied basic research should be conducted to develop more arsenic products with market application prospects and provide effective pathways for arsenic.

## Figures and Tables

**Table 1:** Relationship between arsenic occurrence, speciation, process selection, and final arsenic destination.

Feedstock/Mineral Phase	Representative As Form/State	Key Processing Behavior	Preferred Treatment Route	Typical As Destination/Product
Arsenopyrite, FeAsS	arsenic-bearing sulfide/arsenide; formal As(−I)	Requires oxidation for effective As transfer; As can volatilize during oxidative roasting [18]	Oxidative roasting; oxidative leaching	As_2_O_3_-rich dust; As(III)/As(V)-bearing solution
Realgar, As_4_S_4_	Sulfide; formal As(II)	Readily oxidized to volatile arsenic oxides at elevated temperature	Oxidative roasting	As_2_O_3_/As_4_O_6_ → condensed As_2_O_3_
Orpiment, As_2_S_3_	Sulfide; As(III)	Can be oxidized/volatilized or dissolved in alkaline/sulfide media	Roasting; alkaline/sulfide leaching	As_2_O_3_; thioarsenate solution; As_2_S_3_ intermediate
Enargite, Cu_3_AsS_4_	Cu–As sulfosalt; commonly described using high formal As valence	Cu recovery and As separation are strongly coupled [19]	Controlled roasting/smelting; selective/oxidative leaching	Arsenic-rich dust/solution + Cu-rich product
Arsenic oxides, As_2_O_3_/As_2_O_5_	As(III)/As(V)	Relatively amenable to aqueous dissolution and redox conversion	Hot-water, acidic or alkaline leaching; purification	Refined As_2_O_3_; arsenic-bearing solution; stabilized As(V)
Ferric/calcium arsenates	As(V)	Low volatility; stability and dissolution strongly depend on pH and mineral structure	Acid/alkaline conversion or controlled stabilization	As(V)-bearing solution; ferric arsenate/scorodite residue
Cu–As/Pb–As alloys	Metallic/intermetallic arsenic-bearing phase	Separation can exploit vapor-pressure and phase-partition differences [20]	Vacuum distillation; phase transformation; refining	Arsenic-rich condensate/As_2_O_3_; purified metal phase

**Table 2:** Chemical reactions involved in arsenic removal by multiple-hearth roasting.

No.	Chemical Reaction	Note
1	2As_2_S_3_ + 9O_2_ → 2As_2_O_3_ + 6SO_2_	Oxidation of arsenic sulfide
2	As_4_S_4_ + 7O_2_ → 2As_2_O_3_ + 4SO_2_	Oxidation of realgar
3	2As_2_O_3(s)_ → As_4_O_6(g)_	Sublimation and volatilization of As_2_O_3_
4	As_4_O_6(g)_ → 2As_2_O_3(s)_	Condensation of arsenic-bearing vapor
5	As_2_O_3_ + O_2_ → As_2_O_5_	Further oxidation of As(III)

**Table 3:** Major chemical reactions involved in arsenic removal by rotary-kiln roasting.

No.	Chemical Reaction	Note
6	2As_2_S_3_ + 9O_2_ → 2As_2_O_3_ + 6SO_2_	Oxidation of arsenic sulfide
7	As_4_S_4_ + 7O_2_ → 2As_2_O_3_ + 4SO_2_	Oxidation of realgar
8	2As_2_O_3(s)_ → As_4_O_6(g)_	Sublimation and volatilization of As_2_O_3_
9	As_4_O_6(g)_ → 2As_2_O_3(s)_	Condensation of arsenic-bearing vapor
10	As_2_O_3_ + O_2_ → As_2_O_5_	Further oxidation of As(III)
11	2FeAsS + 5O_2_ → Fe_2_O_3_ + As_2_O_3_ + 2SO_2_ ↑	Oxidation of arsenopyrite
12	Fe_2_O_3_ + As_2_O_5_ → 2FeAsO_4_	Ferric arsenate immobilization
13	3CaO + As_2_O_5_ → Ca_3_(AsO_4_)_2_	Calcium arsenate immobilization
14	4Cu_3_AsS_4_ + 25O_2_ → 12CuO + 2As_2_O_3_ + 16SO_2_	Oxidative roasting of copper–arsenic sulfide

The upward arrow (↑) denotes gas evolution.

**Table 4:** Major chemical reactions involved in arsenic removal by vacuum roasting/distillation.

No.	Chemical Reaction	Note
15	4As_(s)_ → As_4(g)_	Sublimation and volatilization of elemental arsenic
16	As_4(g)_ → 4As_(s)_	Condensation of elemental arsenic vapor
17	2As_2_O_3(s)_ → As_4_O_6(g)_	Sublimation and volatilization of As_2_O_3_
18	As_4_O_6(g)_ → 2As_2_O_3(s)_	Condensation of arsenic-bearing vapor
19	As_2_O_5_ → As_2_O_3_ + O_2_	Thermal decomposition of high-valent arsenic oxide
20	4FeAsS → 4FeS + As_4(g)_	Thermal decomposition of arsenopyrite
21	2As_2_O_3_ + 6C → As_4(g)_ + 6CO	Reductive volatilization of As_2_O_3_
22	2FeAsO_4_ → Fe_2_O_3_ + As_2_O_5_	Thermal decomposition of ferric arsenate

**Table 5:** Major chemical reactions involved in arsenic removal by bath smelting.

No.	Chemical Reaction	Note
23	2As_2_S_3_ + 9O_2_ → 2As_2_O_3_ + 6SO_2_	Oxidation of arsenic sulfide
24	2FeAsS + 5O_2_ → Fe_2_O_3_ + As_2_O_3_ + 2SO_2_	Oxidation of arsenopyrite
25	4As + 3O_2_ → 2As_2_O_3_	Oxidation of elemental arsenic
26	2As_2_O_3(l)_ → As_4_O_6(g)_	Volatilization of As_2_O_3_
27	As_4_O_6(g)_ → 2As_2_O_3(s)_	Condensation of arsenic-bearing vapor
28	Fe_2_O_3_ + As_2_O_5_ → 2FeAsO_4_	Ferric arsenate immobilization
29	3CaO + As_2_O_5_ → Ca_3_(AsO_4_)_2_	Calcium arsenate immobilization

**Table 6:** Quantitative comparison of representative pyrometallurgical arsenic separation technologies.

Technology/Process	Representative Feedstock	Representative Operating Conditions	Residence/Treatment Time	As Separation/Removal Performance	Valuable Metal Recovery/Retention	Main As Destination/Product	Development Status	Technology/Process
Multiple-hearth roasting	Enargite-rich Cu concentrate; representative El Indio feed contained ~23 wt% Cu and ~10 wt% As	14-hearth Nicholas–Herreschoff roaster, 6.5 m diameter; 500–700 °C; O_2_ < 0.5%, essentially neutral roasting	~3 h	Industrial data reported about 94.5% As extraction, with As decreasing from 7.2 wt% in feed to 0.48 wt% in calcine [49]	Cu recovery not explicitly reported; Cu remained principally in calcine	Volatile As species subsequently oxidized/condensed and recovered mainly as As_2_O_3_-rich product/dust	Commercially/industrially operated historical El Indio process	Multiple-hearth roasting
Rotary-kiln roasting	Historical Pb-smelting slag co-treated with ZnO ore	Industrial kiln Φ2.8 m × 44 m; 1100–1200 °C; capacity 120 t d^−1^; coal 0.46–0.53 t t^−1^ feed; kiln speed 0.7–0.95 r min^−1^; negative pressure ~60 Pa	Material residence time NR; each industrial test condition operated stably for 3 d	As volatilization was limited; 47.21–72.48% of total As remained in water-quenched slag	>70% Pb and Zn recovered into Pb–Zn-rich dust	Nonvolatile As mainly converted to Fe–As intermetallic phases and/or immobilized in silicate slag	Industrial-scale demonstration	Rotary-kiln roasting
Vacuum roasting/distillation	Tin anode slime; arsenic-rich Sn-containing polymetallic secondary material	Vacuum distillation: 2 Pa, 1153–1353 K (880–1080 °C); vacuum reduction roasting benchmark: 750 °C, 10 wt% anthracite	30–120 min for vacuum distillation; 3 h for vacuum reduction roasting	At 2 Pa: 84.87–91.90% As removal at 1153 K and 99.43–99.59% at 1353 K; another study obtained 96.97% As removal and residual As 0.53 wt% [37]	Vacuum reduction roasting gave 84.48% Sn recovery; Sn grade increased from 17.70 to 31.41 wt%	Volatile As/As_2_O_3_-rich condensate, while Sn-rich residue is retained	Laboratory-scale; strong potential for high-selectivity refining	Vacuum roasting/distillation
Smelting method	Mixed arsenic-bearing Cu concentrates; arsenic-depleted sublimation residue + Cu concentrate in related smelting study	Oxygen-enriched top-blown smelting at 1180 °C; slag composition/CaO adjusted to enhance As immobilization. Related cyclone smelting: 41–47 kg h^−1^, air excess coefficient 1.1–1.27	NR in top-blown study	Increasing slag As capture caused As content in slag to increase by 20–50%, while As in dust decreased by 10–30% and in matte by 10–20%	Related cyclone-smelting tests achieved 89.73–93.79% Cu recovery to matte; Au recovery 93.72–93.92%	Mainly arsenate-bearing silicate slag under enhanced immobilization conditions; part may still partition to dust/off-gas	Top-blown: experimental validation; related cyclone smelting: pilot-scale, ≤1 t d^−1^	Top-blown bath smelting

**Table 7:** Major chemical reactions involved in acidic leaching for arsenic separation.

No.	Chemical Reaction	Note
30	As_2_O_3_ + 3H_2_O → 2H_3_AsO_3_	Dissolution of As_2_O_3_
31	As_2_O_5_ + 3H_2_O → 2H_3_AsO_4_	Hydration of As_2_O_5_
32	2H_3_AsO_3_ + O_2_ → 2H_3_AsO_4_	Oxidation of As(III)
33	FeAsO_4(s)_ + 3H^+^_(aq)_ → Fe^3+^_(aq)_ + H_3_AsO_4(aq)_	Acid dissolution of ferric arsenate
34	Ca_3(_AsO_4)2(s)_ + 6H^+^_(aq)_ → 3Ca^2+^_(aq)_ + 2H_3_AsO_4(aq)_	Acid dissolution of calcium arsenate
35	Cu_3(_AsO_4)2(s)_ + 6H^+^_(aq)_ → 3Cu^2+^_(aq)_ + 2H_3_AsO_4(aq)_	Acid dissolution of copper arsenate
36	As_2_S_3_ + 7O_2_ + 6H_2_O → 2H_3_AsO_4_ + 3H_2_SO_4_	Oxidative leaching of arsenic sulfide
37	As_2_S_3_ + 6Fe^3+^ + 6H_2_O → 2H_3_AsO_3_ + 3S_(s)_ + 6Fe^2+^ + 6H^+^	Ferric-salt oxidative leaching of arsenic sulfide

**Table 8:** Major chemical reactions involved in alkaline leaching for arsenic separation.

No.	Chemical Reaction	Note
38	As_2_O_3_ + 2NaOH → 2NaAsO_2_ + H_2_O	Alkaline dissolution of As_2_O_3_
39	As_2_O_5_ + 6NaOH → 2Na_3_AsO_4_ + 3H_2_O	Alkaline dissolution of As_2_O_5_
40	FeAsO_4_ + 3NaOH → Na_3_AsO_4_ + Fe(OH)_3(s)_	Alkaline leaching of ferric arsenate
41	Ca_3_(AsO_4_)_2_ + 3Na_2_CO_3_ → 2Na_3_AsO_4_ + 3CaCO_3(s)_	Conversion of calcium arsenate
42	As_2_S_3_ + 6NaOH → Na_3_AsO_3_ + Na_3_AsS_3_ + 3H_2_O	Alkaline dissolution of arsenic sulfide
43	As_2_S_3_ + 12NaOH + 7O_2_ → 2Na_3_AsO_4_ + 3Na_2_SO_4_ + 6H_2_O	Oxidation of arsenic sulfide

**Table 9:** Major chemical reactions involved in hot-water/neutral leaching for arsenic separation.

No.	Chemical Reaction	Note
44	As_2_O_3_ + 3H_2_O → 2H_3_AsO_3_	Hydration and dissolution of As_2_O_3_
45	As_2_O_5_ + 3H_2_O → 2H_3_AsO_4_	Hydration and dissolution of As_2_O_5_
46	2H_3_AsO_3_ + O_2_ → 2H_3_AsO_4_	Oxidation of As(III)
47	H_3_AsO_3_ + H_2_O_2_ → H_3_AsO_4_ + H_2_O	Oxidation by hydrogen peroxide
48	NaAsO_2_ → Na^+^ +AsO_2_^−^	Dissolution of arsenite
49	Na_3_AsO_4_ → 3Na^+^ +AsO_4_^3−^	Dissolution of arsenate
50	H_3_AsO_4_ ⇌ H_2_AsO_4_^−^ + H^+^	First dissociation of As(V)
51	H_2_AsO_4_^−^ ⇌ HAsO_4_^2−^ + H^+^	Second dissociation of As(V)

**Table 10:** Major chemical reactions involved in inorganic-salt leaching for arsenic separation.

No.	Chemical Reaction	Note
52	As_2_O_3_ + Na_2_CO_3_ → 2NaAsO_2_ + CO_2_	Carbonate leaching of As_2_O_3_
53	As_2_O_5_ + 3Na_2_CO_3_ → 2Na_3_AsO_4_ + 3CO_2_	Carbonate leaching of As_2_O_5_
54	Ca_3_(AsO_4_)_2_ + 3Na_2_CO_3_ → 2Na_3_AsO_4_ + 3CaCO_3(s)_	Conversion leaching of calcium arsenate
55	As_2_S_3_ + 3Na_2_S → 2Na_3_AsS_3_	Sulfide-salt leaching of arsenic sulfide
56	H_3_AsO_3_ + 2FeCl_3_ + H_2_O → H_3_AsO_4_ + 2FeCl_2_ + 2HCl	Ferric-salt oxidation
57	As_2_S_3_ + 6FeCl_3_ + 6H_2_O → 2H_3_AsO_3_ + 3S_(s)_ + 6FeCl_2_ + 6HCl	Ferric-salt leaching of arsenic sulfide

**Table 11:** Major chemical reactions involved in arsenic removal by lime neutralization and precipitation.

No.	Chemical Reaction	Note
58	CaO + H_2_O → Ca(OH)_2_	Lime hydration
59	Ca(OH)_2_ → Ca^2+^ + 2OH^−^	Dissociation of lime slurry
60	H^+^ + OH^−^ → H_2_O	Acid–base neutralization
61	2H_3_AsO_4_ + 3Ca(OH)_2_ → Ca_3_(AsO_4_)_2(s)_ + 6H_2_O	Calcium arsenate precipitation
62	2H_3_AsO_3_ + 3Ca(OH)_2_ → Ca_3_(AsO_3_)_2(s)_ + 6H_2_O	Calcium arsenite precipitation
63	Fe^3+^ + 3OH^−^ → Fe(OH)_3(s)_	Ferric coprecipitation

**Table 12:** Major chemical reactions involved in arsenic removal by sulfide precipitation.

No.	Chemical Reaction	Note
64	Na_2_S + 2H^+^ → H_2_S + 2Na^+^	Acidification of sulfide reagent
65	2H_3_AsO_3_ + 3H_2_S → As_2_S_3(s)_ + 6H_2_O	As(III) sulfide precipitation
66	2H_3_AsO_4_ + 5H_2_S → As_2_S_3(s)_ + 2S_(s)_ + 8H_2_O	Reductive sulfidation of As(V)
67	2As^3+^ + 3S^2−^ → As_2_S_3(s)_	Formal arsenic-ion sulfide precipitation reaction
68	2Na_3_AsS_3_ + 6H^+^ → As_2_S_3(s)_ + 3H_2_S_(g)_ + 6Na^+^	Acidification precipitation of thioarsenate

**Table 13:** Major chemical reactions involved in arsenic removal by ferric coprecipitation (lime–ferric-salt process).

No.	Chemical Reaction	Note
69	CaO + H_2_O → Ca(OH)_2_	Lime hydration
70	Fe^3+^ + 3OH^−^ → Fe(OH)_3 (s)_	Ferric-salt hydrolysis
71	2H_3_AsO_3_ + O_2_ → 2H_3_AsO_4_	Oxidation of As(III)
72	Fe^3+^ + H_2_AsO_4_^−^ + 2H_2_O → FeAsO_4_·2H_2_O_(s)_ + 2H^+^	Ferric arsenate precipitation
73	≡FeOH + H_2_AsO_4_^−^ → ≡FeHAsO_4_^−^ + H_2_O	Iron-hydroxyl surface adsorption
74	2H_3_AsO_4_ + 3Ca(OH)_2_ → Ca_3_(AsO_4_)_2(s)_ + 6H_2_O	Calcium arsenate precipitation

Note: ≡FeOH denotes a surface hydroxyl group on the adsorbent.

**Table 14:** Major chemical reactions involved in arsenic removal by scorodite crystallization.

No.	Chemical Reaction	Note
75	2H_3_AsO_3_ + O_2_ → 2H_3_AsO_4_	Oxidation of As(III)
76	4Fe^2+^ + O_2_ + 4H ^+^ → 4Fe^3+^ + 2H_2_O	Oxidation of Fe(II)
77	Fe^3+^ + H_3_AsO_4_ + 2H_2_O → FeAsO_4_·2H_2_O_(s)_ + 3H^+^	Scorodite crystallization
78	Fe^3+^ + H_2_AsO_4_^−^ + 2H_2_O → FeAsO_4_·2H_2_O_(s)_ + 2H^+^	Arsenate crystallization
79	Fe^3+^ + 3H_2_O → Fe(OH)_3(s)_ + 3H^+^	Ferric-salt hydrolysis side reaction

**Table 15:** Major reactions involved in ion exchange and adsorption for arsenic removal.

No.	Chemical Reaction	Note
80	2H_3_AsO_3_ + O_2_ → 2H_3_AsO_4_	Oxidation of As(III)
81	H_3_AsO_4_ ⇌ H_2_AsO_4_^−^ + H^+^	Dissociation of As(V)
82	H_2_AsO_4_^−^ ⇌ HAsO_4_^2−^ + H^+^	Arsenate speciation change
83	≡FeOH + H_2_AsO_4_^−^ → ≡FeHAsO_4_^−^ + H_2_O	Adsorption on iron oxide
84	≡AlOH + H_2_AsO_4_^−^ → ≡AlHAsO_4_^−^ + H_2_O	Adsorption on alumina
85	R–Cl + H_2_AsO_4_^−^ → R–H_2_AsO_4_ + Cl^−^	Anion-exchange reaction

Note: ≡FeOH and ≡AlOH denote surface hydroxyl groups on the adsorbent.

**Table 16:** Major extraction reactions or equilibria involved in solvent extraction for arsenic separation.

No.	Chemical Reaction/Equilibrium	Note
86	H_3_AsO_3_ + 3HCl ⇌ AsCl_3_ + 3H_2_O	Chloride complexation of As(III)
87	AsCl_3_(aq) + nL_(org)_ ⇌ AsCl_3_·nL_(org)_	Extraction of neutral complex
88	R_3_N_(org)_ + H^+^ + Cl^−^ ⇌ R_3_NH^+^ Cl^−^_(org)_	Protonation of amine extractant
89	R_3_NH^+^ Cl^−^_(org)_ + H_2_AsO_4_^−^ ⇌ R_3_NH ^+^ H_2_AsO_4_^−^_(org)_ + Cl^−^	Anion-exchange extraction of As(V)
90	R_3_NH^+^ H_2_AsO_4_^−^_(org)_ + OH^−^ ⇌ R_3_N_(org)_ + H_2_AsO_4_^−^ + H_2_O	Arsenic stripping and extractant regeneration

Note: L_(org)_ denotes a neutral extractant, such as TBP or TOPO; R_3_N denotes a tertiary amine extractant.

**Table 18:** Major chemical reactions involved in the roasting–leaching process.

No.	Chemical Reaction	Note
91	2As_2_S_3_ + 9O_2_ → 2As_2_O_3_ + 6SO_2(g)_	Oxidation of arsenic sulfide
92	2FeAsS + 5O_2_ → Fe_2_O_3_ + As_2_O_3_ + 2SO_2(g)_	Oxidation of arsenopyrite
93	2As_2_O_3(s)_ → As_4_O_6(g)_	Volatilization of As_2_O_3_
94	As_4_O_6(g)_ → 2As_2_O_3(s)_	Condensation of arsenic-bearing vapor
95	As_2_O_3_ + 3H_2_O → 2H_3_AsO_3_	Hydration leaching of As_2_O_3_
96	FeAsO_4_ + 3H^+^ → Fe^3+^ + H_3_AsO_4_	Acid leaching of ferric arsenate
97	2H_3_AsO_3_ + O_2_ → 2H_3_AsO_4_	Oxidation of As(III)

**Table 19:** Major chemical reactions involved in the vacuum-distillation–leaching process.

No.	Chemical Reaction	Note
98	2As_2_O_3(s)_ → As_4_O_6(g)_	Vacuum volatilization of As_2_O_3_
99	4As_(s)_ → As_4(g)_	Vacuum volatilization of elemental arsenic
100	As_4_O_6(g)_ → 2As_2_O_3(s)_	Condensation of arsenic-oxide vapor
101	As_4(g)_ → 4As_(s)_	Condensation of elemental arsenic vapor
102	As_2_O_3_ + 3H_2_O → 2H_3_AsO_3_	Hydration leaching of As_2_O_3_
103	2H_3_AsO_3_ + O_2_ → 2H_3_AsO_4_	Oxidation of As(III)
104	CuO + H_2_SO_4_ → CuSO_4_ + H_2_O	Acid leaching of copper oxide
105	ZnO + H_2_SO_4_ → ZnSO_4_ + H_2_O	Acid leaching of zinc oxide

**Table 20:** Major chemical reactions involved in the smelting–leaching–electrowinning process.

No.	Chemical Reaction	Note
106	2CuFeS_2_ + 4O_2_ → Cu_2_S + 2FeO + 3SO_2(g)_	Oxidation of copper sulfide
107	FeO + SiO_2_ → FeSiO_3_	Silicate slag-forming reaction
108	4As + 3O_2_ → 2As_2_O_3_	Oxidation of arsenic
109	2As_2_O_3(s/L)_ → As_4_O_6(g)_	Volatilization of arsenic oxide
110	Fe_2_O_3_ + As_2_O_5_ → 2FeAsO_4_	Ferric arsenate immobilization
111	CuO + H_2_SO_4_ → CuSO_4_ + H_2_O	Acid leaching of copper oxide
112	2H_3_AsO_3_ + O_2_ → 2H_3_AsO_4_	Oxidation of As(III)
113	Fe^3+^ + H_3_AsO_4_ + 2H_2_O → FeAsO_4_·2H_2_O_(s)_ + 3H^+^	Ferric arsenate precipitation
114	2CuSO_4_ + 2H_2_O → 2Cu + O_2(g)_ + 2H_2_SO_4_	Copper electrowinning

**Table 21:** Major chemical reactions involved in the high-temperature filtration–hydrometallurgical process.

No.	Chemical Reaction	Note
115	2As_2_S_3_ + 9O_2_ → 2As_2_O_3_ + 6SO_2(g)_	Oxidation of arsenic sulfide
116	4As + 3O_2_ → 2As_2_O_3_	Oxidation of elemental arsenic
117	2As_2_O_3(s/L)_ → As_4_O_6(g)_	Volatilization of arsenic oxide
118	As_4_O_6(g)_ → 2As_2_O_3(s)_	Condensation of arsenic-bearing vapor
119	As_2_O_3_ + 3H_2_O → 2H_3_AsO_3_	Hydration leaching of As_2_O_3_
120	2H_3_AsO_3_ + O_2_ → 2H_3_AsO_4_	Oxidation of As(III)
121	Fe^3+^ + H_3_AsO_4_ + 2H_2_O → FeAsO_4_·2H_2_O_(s)_ + 3H^+^	Scorodite crystallization
122	CuO + H_2_SO_4_ → CuSO_4_ + H_2_O	Acid leaching of valuable metal

## Data Availability

No new data were created or analyzed in this study. Data sharing is not applicable to this article.
